# Exotic Fruit Peel Extracts as Green Resources for Bioactive Silver-Based Materials with Plant Protection Potential

**DOI:** 10.3390/ma19163492

**Published:** 2026-08-18

**Authors:** Oana-Alexandra Luțu, Bianca-Maria Neagoe, Camelia Ungureanu, Codruța Mihaela Dobrescu, Loredana Elena Vijan, Carmen Mihaela Topală, Georgiana Cîrstea, Aurelian Denis Negrea, Nicoleta Anca Șuțan, Alina Păunescu, Elena Simona Pisculungeanu, Constanța Bucăloiu, Liliana Cristina Soare

**Affiliations:** 1 Faculty of Sciences, Physical Education and Computer Science, Pitesti University Centre, National University of Science and Technology Politehnica Bucharest, 1st Targu din Vale Str., 110040 Pitesti, Romania; oana.draghiceanu@upb.ro (O.-A.L.); bianca.smarandoiu@stud.sefi.upb.ro (B.-M.N.); codruta.dobrescu@upb.ro (C.M.D.); loredana.vijan@upb.ro (L.E.V.); carmen.topala@upb.ro (C.M.T.); nicoleta_anca.sutan@upb.ro (N.A.Ș.); alina.paunescu@upb.ro (A.P.); pisculungeanue@gmail.com (E.S.P.); mirelabucaloiu3@gmail.com (C.B.); liliana.soare@upb.ro (L.C.S.); 2 Faculty of Chemical Engineering and Biotechnology, National University of Science and Technology Politehnica Bucharest, 313 Splaiul Independentei, Sector 6, 060042 Bucharest, Romania; 3 Regional Research and Development Center for Innovative Materials, Processes and Products for the Automotive Industry (CRC&D-Auto), Pitesti University Centre, National University of Science and Technology Politehnica Bucharest, 1st Targu din Vale Str., 110040 Pitesti, Romania; georgiana.cirstea93@upb.ro (G.C.); aurelian.negrea@upb.ro (A.D.N.)

**Keywords:** circular economy, agro-food waste valorization, fruit peel extracts, green synthesis, silver nanoparticles, antifungal activity, phytotoxicity, sustainable agriculture

## Abstract

Fruit peels represent an abundant source of bioactive compounds with potential applications in green nanotechnology and plant protection. This study comparatively evaluated ethanolic extracts obtained from pomegranate (*Punica granatum* L.), orange (*Citrus sinensis* Osbeck), and persimmon (*Diospyros kaki* L.) peels and the corresponding silver nanoparticle (AgNP)-containing phytosynthesis preparations. The extracts were characterized for phytochemical and antioxidant properties and used for AgNP phytosynthesis, followed by UV-Vis, FTIR, BF-STEM, and EDS characterization. Their effects on *Triticum aestivum* L., *Zea mays* L., and *Cucumis sativus* L., as well as their antifungal activity against selected fungal strains, were comparatively assessed. Pomegranate peel extract showed the highest total phenolic content (147.52 mg g^−1^ fw) and the strongest antifungal activity against *Penicillium hirsutum*, with an inhibition zone of 30 mm, followed by orange (27 mm) and persimmon (17.33 mm) peel extracts. The AgNP-containing preparations generally produced lower inhibition zones than the corresponding crude extracts. Antioxidant activity also decreased following phytosynthesis, with the most pronounced reduction observed for the orange peel system (90.72% to 35.17%). Plant responses were species- and treatment-dependent, with maize showing the lowest overall sensitivity to the investigated treatments. Overall, the results demonstrate that conversion of fruit peel extracts into AgNP-containing preparations does not necessarily enhance their biological activity and emphasize the importance of considering both the phytochemical characteristics of the starting extracts and the biological target when developing fruit-waste-derived systems for potential plant-protection applications.

## 1. Introduction

In recent years, the valorization of agri-food by-products has become an important direction within the circular economy, as it allows the reduction of the amount of organic waste and its transformation into value-added resources [1,2]. Fruit peels, frequently considered waste resulting from household consumption or the food industry, contain significant amounts of bioactive compounds, such as polyphenols, flavonoids, tannins, organic acids, natural pigments and essential oils. These compounds can exhibit antioxidant, antimicrobial and antifungal activity, which gives them potential for use in agriculture, plant protection and obtaining functional materials. Thus, the use of fruit peels is not only a solution for reducing the environmental impact of vegetable waste, but also a sustainable strategy for obtaining natural products with biotechnological applicability [1,2].

However, not every type of fruit peel can be used directly in such applications. The plant raw material must meet certain safety and quality criteria, as peels can accumulate pesticides, heavy metals, microbiological contaminants, or residues resulting from post-harvest treatments [3,4]. For this reason, the selection of peels must be carried out taking into account the origin of the fruit, the degree of freshness, the absence of visible deterioration, the cleaning method, and the processing conditions. In addition, for agricultural applications or for the green synthesis of nanoparticles, it is important that the extracts obtained have a relatively rich composition in reducing and stabilizing compounds, but also a low level of chemical or biological contamination [3,4].

We selected pomegranate (*Punica granatum* L.), orange (*Citrus sinensis* Osbeck), and persimmon (*Diospyros kaki* L.) peels based on their rich composition in secondary metabolites with biological activity. Pomegranate peel is known for its high content of polyphenols, hydrolyzable tannins, flavonoids, and phenolic acids, compounds associated with antioxidant and antimicrobial activity [5]. Kaki peel also contains phenolic compounds, carotenoids, and tannins, which may contribute to biological effects on microorganisms and physiological processes of plants [6]. Orange peel is an important source of flavonoids, ascorbic acid, phenolic compounds, and specific constituents of essential oils, such as limonene, known for its antimicrobial potential [7,8].

Accordingly, these three types of peels represent interesting plant sources both for testing antifungal activity on phytopathogenic strains and for their use in the phytosynthesis of silver nanoparticles. Phenolic compounds and other reducing metabolites in the extracts can participate in the reduction of silver ions to metallic silver, while other organic molecules can act as stabilizing agents of the formed nanoparticles [9,10]. Thus, plant extracts can have a dual role: a source of bioactive compounds and a natural environment for the green synthesis of silver nanoparticles (AgNPs). AgNPs are intensively studied due to their antimicrobial and antifungal properties and potential use in agriculture, medicine, biotechnology, and environmental protection. Conventional synthesis methods may involve aggressive chemical reagents, toxic solvents, or working conditions with a negative impact on the environment. In this context, green synthesis of nanoparticles, using plant extracts, represents a sustainable, safer, and more environmentally friendly alternative [11,12]. Phytosynthesis of AgNPs allows the use of natural compounds from extracts as reducing agents and stabilizers, eliminating or reducing the need for additional chemicals [11,12]. In addition, nanoparticles obtained by green methods can be coated on the surface with bioactive molecules from the plant extract, which can contribute to improving stability, biocompatibility, and biological activity. In the case of agricultural applications, such nanoparticles can be investigated as agents with antifungal potential, as germination stimulants, or as alternative treatments to reduce phytopathogen pressure. Obtaining silver nanoparticles based on fruit peel extracts aims to combine the bioactive effect of plant compounds with the antimicrobial properties specific to silver. Thus, a complex action system can be obtained, in which the plant extract contributes through its content of polyphenols, flavonoids, and other metabolites, and the silver nanoparticles can amplify the antifungal or antimicrobial effect.

While plant extracts and nanoparticles obtained by green synthesis are considered more sustainable alternatives, the evaluation of their toxicity is essential before proposing agricultural applications. A natural compound is not automatically free of negative effects, and nanoparticles can have different effects depending on concentration, size, stability, and the type of organism exposed [13]. In agriculture, an effective treatment against phytopathogens must be selective: inhibit the growth of harmful microorganisms, but not adversely affect germination, plant growth, or their metabolism. For this reason, phytotoxicity testing on *Triticum aestivum* L., *Zea mays* L. and *Cucumis sativus* L. is justified, as these species represent relevant plant models for evaluating the impact of extracts on crops [14,15]. Measuring the development of axial organs, pigment content and total phenolic content allows assessing the effect of treatments on plant growth, photosynthesis and antioxidant response. Thus, the study not only aims to identify extracts or nanoparticles with antifungal effect, but also to establish a balance between biological efficiency and safety for plants.

The microorganisms tested—*Aspergillus niger*, *Botrytis cinerea*, *Fusarium oxysporum* and *Penicillium* spp.—are important because they include species or genera frequently involved in spoilage of plant products, reduction of crop quality and significant economic losses in agriculture and the food industry. These fungi can affect plants at different stages of development or contaminate products after harvest, contributing to the qualitative and quantitative deterioration of plant raw materials [16]. *Botrytis cinerea* is associated with gray rot and affects many horticultural crops, especially in high humidity conditions. *Fusarium oxysporum* is known for its ability to cause vascular diseases and wilting in plants, being difficult to control due to its persistence in the soil. *Aspergillus niger* and *Penicillium* spp. are frequently involved in the spoilage of stored food and agricultural products, and some species can produce toxic metabolites or significantly reduce the quality of the products. For these reasons, their control by alternative methods, based on natural extracts and nanoparticles obtained by green synthesis, represents a direction of interest for sustainable plant protection [17,18,19].

In this context, the exploitation of exotic fruit peels as a source of bioactive compounds and reducing agents for the green synthesis of silver nanoparticles represents a promising approach for the development of sustainable solutions for plant protection.

Although the phytosynthesis of AgNPs using individual fruit peel extracts has been previously reported, comparative studies integrating different fruit peel-derived systems under the same experimental framework remain limited. In the present study, pomegranate, orange, and persimmon peel extracts and their corresponding AgNP-containing preparations were comparatively evaluated by integrating phytochemical and physicochemical characterization with plant-response and antifungal assessments. This approach was intended to examine whether the conversion of fruit peel extracts into AgNP-containing systems provides consistent changes in their biological performance.

## 2. Materials and Methods

### 2.1. Obtaining the Extracts and AgNPs by Phytosynthesis

The extracts were obtained from the epicarp of *Punica granatum* L. (R), *Diospyros kaki* L. (K), and *Citrus sinensis* Osbeck (P), the fruits coming from a local trader. The solvent used was 96% ethanol, with a ratio of plant material to solvent of 1:10, and the mixture was left at room temperature for 7 days and then filtered. After filtering, the content of polyphenols, which are the reducing agents of Ag ions [20], was determined, and the UV-Vis spectrum was plotted.

For the phytosynthesis of AgNPs, the ethanolic extracts were used directly, without solvent evaporation, and mixed with an aqueous 10^−3^ M (1 mM) AgNO_3_ solution at a 1:1 (*v*/*v*) ratio; thus, the phytosynthesis reaction proceeded in a hydroalcoholic medium.

The phytosynthesis reaction was carried out at room temperature (approximately 20 ± 2 °C) for 60 h, without pH adjustment. The final pH of the reaction mixture was not recorded.

The resulting phytosynthesis preparations were used as obtained, without separation or purification of the nanoparticulate fraction. Therefore, these samples contained the phytosynthesized AgNPs together with the remaining extract-derived compounds and potentially residual ionic silver.

### 2.2. Total Phenol Content (TPC) of Extracts and AgNP Mixtures

To determine the TPC, the Singleton and Rossi method (1965) modified by Orţan et al. (2015) [21] was used. A 1 mL sample (100-fold diluted) + 5 mL of Folin–Ciocalteu reagent soil was left for 3–8 min; then, 4 mL of Na_2_CO_3_ was added, and the mixture was left at room temperature for 60 min, after which the wavelength was measured at 765 nm (T70+ spectrophotometer—PG Instruments, Leicestershire, UK). The results were interpreted with a curve obtained with different gallic acid solutions.

### 2.3. UV-Vis Analysis

The formation and stability of nanoparticles were examined using the PerkinElmer Lambda 25 UV-Vis Spectrophotometer (PerkinElmer, Inc., Waltham, MA, USA) in the range of 200–600 nm for Ag nanoparticles [22], using a quartz cuvette with an optical path of 10 mm.

### 2.4. BF-STEM—EDS Analysis of AgNP Mixtures

The examination and characterization of the silver nanoparticle functionalized mixtures were performed by bright-field scanning transmission electron microscopy (BF-STEM) and energy-dispersive X-ray spectroscopy (EDS) (Hitachi SU8230 scanning electron microscope, Tokyo, Japan). As a first step in the SEM confirmation of the phytosynthesis of silver nanoparticles, an aliquot of each sample was applied to conductive carbon tape and kept in a desiccator for 24 h for complete evaporation of the solvent. Previously, the samples were subjected to acoustic cavitation in an ultrasonic bath to reduce the tendency of silver nanoparticles to agglomerate. For the size analysis of the phytosynthesized nanoparticles, an aliquot of each sample was transferred into Eppendorf tubes and subjected to acoustic cavitation using the Vial Tweeter sonotrode connected to the Hielscher UP200St ultrasonic system (Teltow, Germany). This procedure was chosen to ensure a uniform dispersion of the metal nanoparticles. Subsequently, one drop of each suspension was applied to carbon film-coated copper grids, which were then kept in a desiccator for 24 h for complete evaporation of the solvent.

### 2.5. Analysis of the AgNP Size Distribution

The particle size distribution was determined by processing the BF-STEM micrographs using ImageJ software (version 1.54g, National Institutes of Health, Bethesda, MD, USA). 

### 2.6. ATR-FTIR Analysis of Extracts and AgNP Mixtures

ATR-FTIR spectra were recorded using a JASCO 6700 FTIR spectrometer equipped with a Pike Technologies diamond ATR accessory (Pike Technologies, Madison, WI, USA) and controlled with Spectra Manager II software, version 2. For each sample, three spectra were collected at a resolution of 4 cm^−1^ with 32 scans per spectrum, in the spectral range 4000–400 cm^−1^.

### 2.7. Determination of Antioxidant Activity (DPPH Method) of Extracts and AgNP Mixtures

From each sample, a volume of 0.25 mL was taken, which was transferred to a test tube, to which 1.75 mL of DPPH solution was added. The mixtures were incubated in the dark, at room temperature, for 30 min, and the absorbance was subsequently determined spectrophotometrically, using an Ocean Optics HR 2000+ spectrophotometer at a wavelength of 517 nm (T70+ spectrophotometer—PG Instruments, Leicestershire, UK).

The percentage of DPPH inhibition (PI) was calculated according to the formula:

PI = [(A0 − A1)/A0] × 100,

where A0—absorbance of the control sample (DPPH + EtOH);

A1—absorbance of the sample (DPPH + extract) [23].

### 2.8. Phytotoxicity Tests of Extracts and AgNP Mixtures

The toxicity of the extracts was tested against 3 species of cultivated plants: *Triticum aestivum* L., *Zea mays* L. (corn), and *Cucumis sativus* L. (cucumber). *Triticum aestivum* (Trivale variety) and *Zea mays* (Amurg variety) seeds were provided by the Agricultural Research and Development Station Piteşti—Albota and the National Institute for Agricultural Research and Development Fundulea, respectively, and *Cucumis sativus* seeds were bought commercially.

The seed hydration and immersion in the test solution took one hour each. After immersion in the test solution, the seeds were placed in Petri dishes (borosilicate glass 90 mm × 15 mm, Novarli, Benešov, Czech Republic) on filter paper (Whatman, O 90 mm, Cole-Parmer, Vernon Hills, IL, USA) and watered periodically with distilled water. The test solution was represented by the extracts diluted 10 and 100 times, respectively (Table 1). For the control, only distilled water was used, and in the case of the ethanol variants, 96% ethanol was used.

For each variant, 10 seeds from each plant species were used, and the experiment had 3 repetitions. Seedling root and stem measurements were performed 7 days after the initiation of the experiment. Root and stem length were determined using graph paper, and a balance (RADWAG WTC 600, Radom, Poland) was used for seedling wet weight. Subsequently, the seedlings were placed in the soil, and after a period of 9 and 13 days, respectively, the content of assimilatory pigments [24] and the content of polyphenols from seedling leaves were determined.

The statistical processing of the obtained data was performed with the IBM SPSS Statistics 23 program: the mean and standard error were calculated, and the means were compared with the Duncan test.

### 2.9. Determination of Assimilatory Pigment Content

An amount of 0.1 g of seedling leaves was weighed and ground in a mortar in 10 mL of pure acetone solution, with sand grains (for more efficient trituration), until the leaf material discolored. The extract was filtered into an Erlenmeyer flask using a funnel and filter paper. One cuvette of the spectrophotometer was filled with the blank solution (acetone), and the other with the extract [23]. The absorbance of the sample was read at three wavelengths: 440 nm, 646 nm, and 662 nm (T70+ spectrophotometer—PG Instruments, Leicestershire, UK). To find the concentrations, the absorbance values obtained were introduced into the following equations [25]:

Chlorophyll *a* (mg) = 9.78 × A662 − 0.99 × A646 Chlorophyll *b* (mg) = 21.4 × A646 − 4.65 × A662 Carotenoids (mg) = 4.69 × A440 − 0.267 (Chlorophyll *a* + Chlorophyll *b*)

where Ax = absorbance at wavelength x nm.

### 2.10. Antifungal Assay

The antifungal assay of plant extracts and silver nanoparticles obtained by phytosynthesis was evaluated by the agar diffusion method. The tests were performed on Potato Dextrose Agar (PDA) medium, using *Aspergillus niger* ATCC 15475, *Fusarium oxysporum* ATCC 48112, *Penicillium hirsutum* ATCC 52323, and *Botrytis cinerea* ATCC 11542. The fungal inoculum was obtained by scraping the developed cultures and suspending the biomass in sterile water. The suspensions thus obtained were distributed uniformly on the surface of the solidified PDA medium. After inoculation, sterile wells with a diameter of 6 mm were made in the agar. Moreover, 50 µL of the analyzed samples, represented by the ethanolic extracts and the corresponding AgNP-containing phytosynthesis preparations, was introduced into each well. Before use, the samples were homogenized by vortexing. The plates were incubated at 28 °C for 72 h. Antifungal activity was assessed by measuring the total diameter of the inhibition zones formed around the wells using a graduated ruler. The ethanol used to obtain the extracts was tested separately and used as a negative control. All determinations were performed in triplicate, and the results were expressed as mean ± standard deviation. Differences between treatments were evaluated by one-way ANOVA, followed by the post hoc Tukey HSD test. Differences were considered statistically significant for *p* values < 0.05.

## 3. Results

### 3.1. TPC and UV-Vis Analysis of Extracts and AgNP-Containing Phytosynthesis Preparations

**Figure 1:**
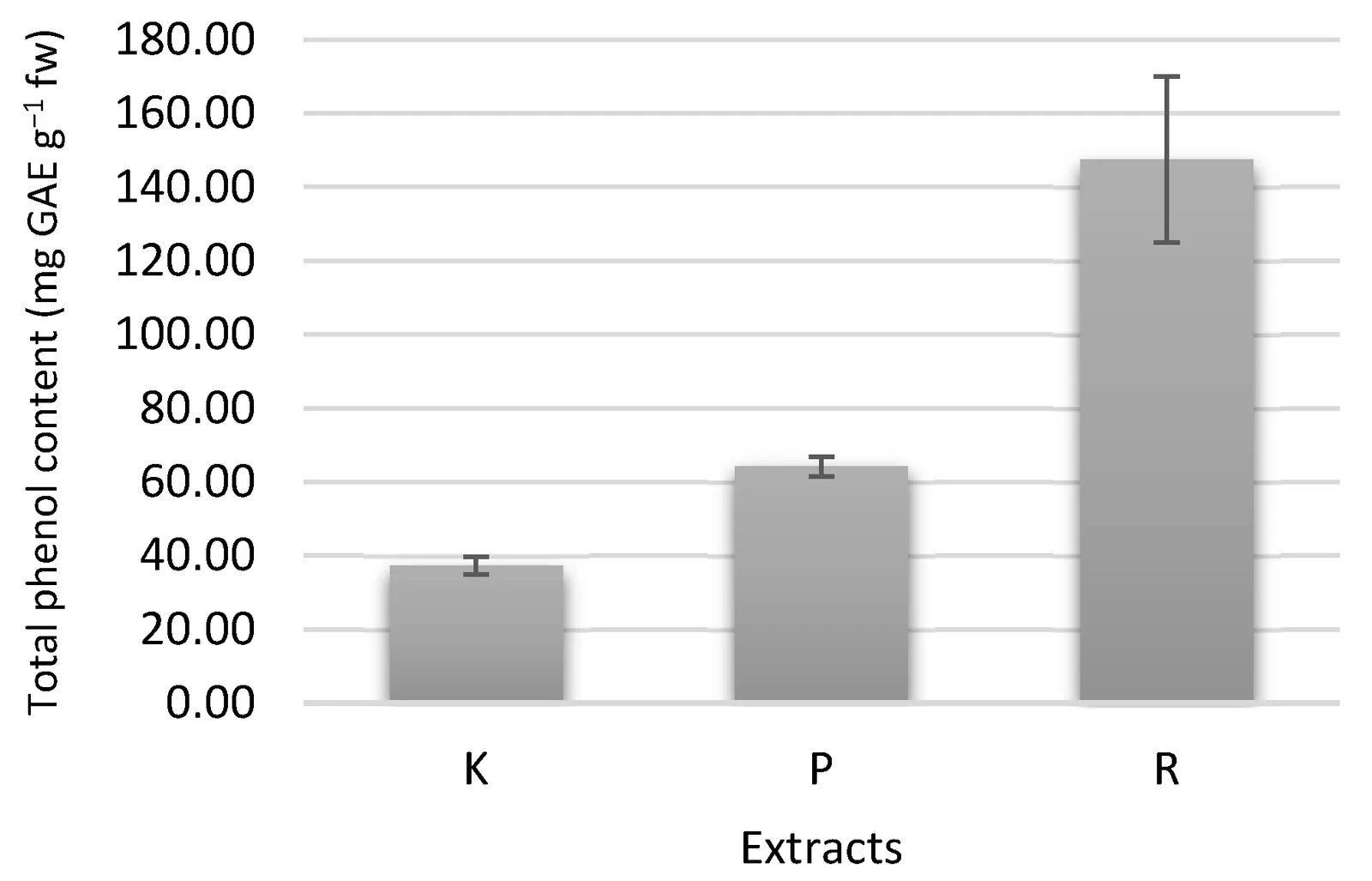
Total phenol content in extracts obtained from the peel of *Punica granatum* L. (R), *Diospyros kaki* L. (K), and *Citrus sinensis* Osbeck (P).

**Figure 2:**
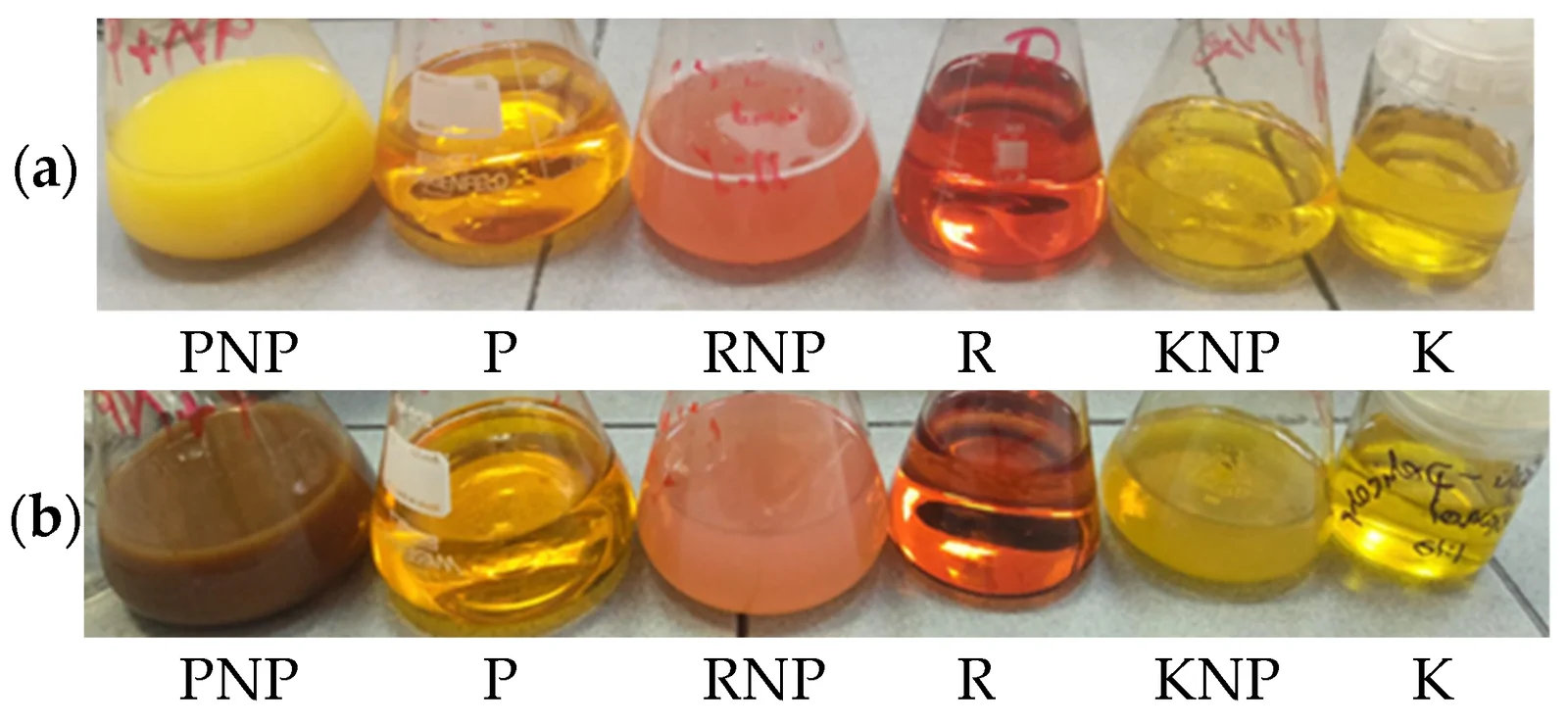
Color change of extracts after nanoparticle phytosynthesis: (**a**) on the first day; (**b**) after three days.

Total phenol content in the pomegranate peel ethanolic extract (R) was greater than that in the orange peel extract (P) and persimmon extract (K) (Figure 1). The macroscopic analysis of the extracts and mixtures showed a color change after silver nitrate was added and nanoparticle phytosynthesis, evident on the first day in the case of pomegranate (from red to pale pink) and orange (from yellow to pale yellow) extracts. The color intensity of the orange mixture increased with the incubation time, and the yellow solution turned dark brown on the third day, which may be due to the increased concentration of nanoparticles (Figure 2). The high phenol content of pomegranate extract (R) helps to rapidly reduce the silver metal ions to silver nanoparticles, which is visible through the extract color change.

**Figure 3:**
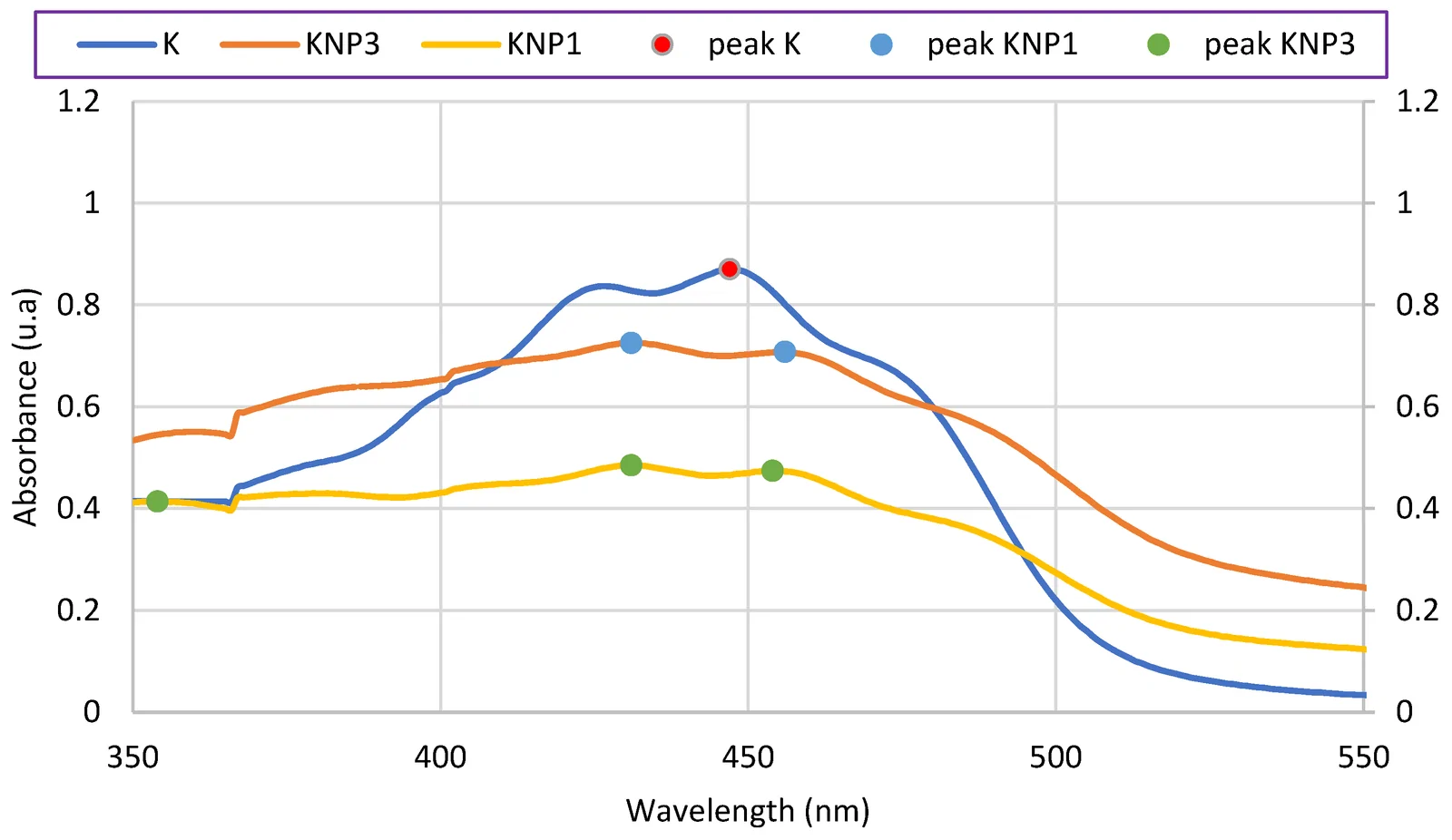
UV-Vis spectra of *Diospyros kaki* L. extract (K) and *AgNP-containing phytosynthesis preparations* on the first day (KNP1) and on the third day (KNP3), and peaks for extract (peak K) and mixture (peak KNP1, peak KNP3).

The *Diospyros kaki* L. extract (K) showed 3 peaks at wavelengths 235, 315 and 447 nm. In the nanoparticle variant (KNP), peaks at wavelengths 235 and 315 nm also appeared, as well as two new peaks at 431 and 455 nm—which increased in intensity from 0.485 and 0.474 on the first day to 0.725 and 0.708 on the third day (Figure 3).

**Figure 4:**
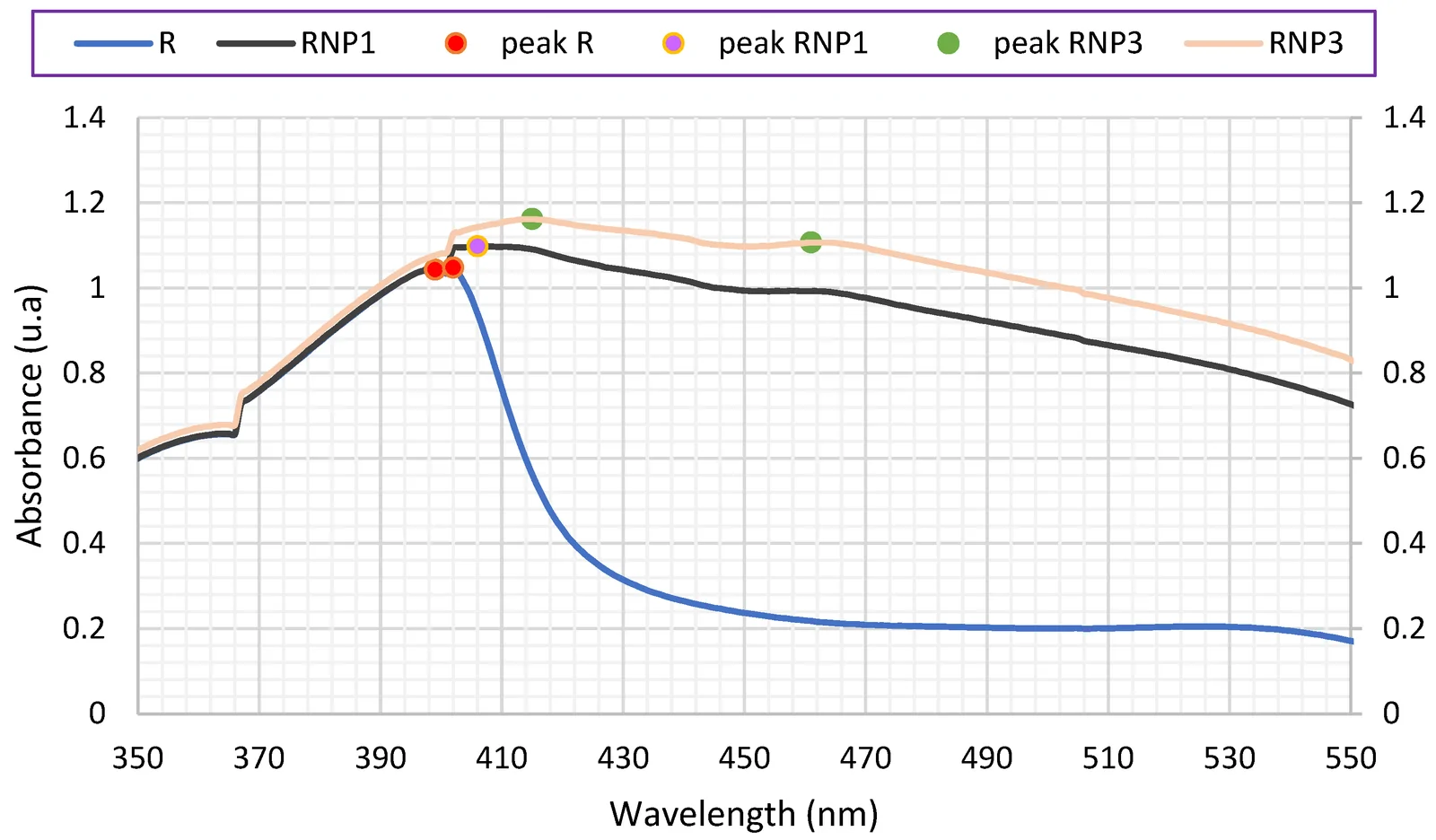
UV-Vis spectra of *Punica granatum* L. extract (R) and *AgNP-containing phytosynthesis preparations* on the first day (RNP1) and on the third day (RNP3), and peaks for extract (peak R) and mixture (peak RNP1, peak RNP3).

In the case of the *Punica granatum* L. extract (R), peaks were recorded at wavelengths 235, 320 and 402 nm. After the addition of AgNO_3_ solution, a slight shift of the peak at 406 nm and an increase in intensity were observed, and on the third day two new peaks appeared at 415 nm and 461 nm (Figure 4).

**Figure 5:**
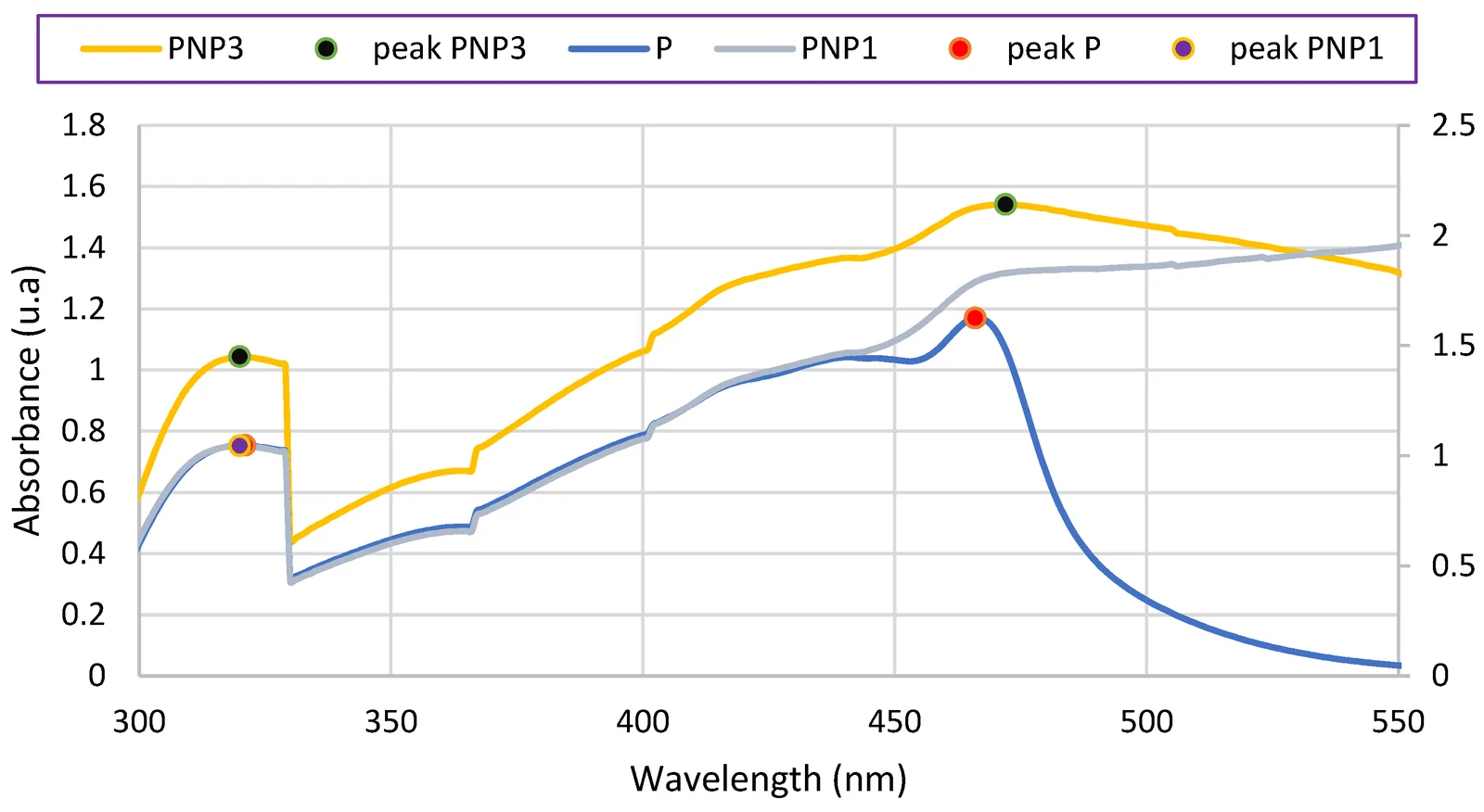
UV-Vis spectra of *Citrus sinensis* Osbeck extract (P) and *AgNP-containing phytosynthesis preparations* on the first day (PNP1) and on the third day (PNP3), and peaks for extract (peak P) and mixture (peak PNP1, peak PNP3).

The *Citrus sinensis* Osbeck extract (P) with nanoparticles was diluted (1:10) on the third day to be analyzed. In Figure 5, in addition to the peaks at 235 and 320 nm, a shift of the peak from 466 to 472 nm can be observed. The peaks at 235 and 315–320 nm indicate the existence of bioactive compounds, especially polyphenols and flavonoids and hydroxycinnamic acids, respectively.

### 3.2. ATR-FTIR Analysis of Extracts and AgNP-Containing Phytosynthesis Preparations

ATR-FTIR analysis was performed to assess the involvement of phytochemical functional groups in AgNP formation and stabilization. For the ethanolic extract of *Diospyros kaki* (K), a broad absorption band centered at 3264 cm^−1^ was assigned to O–H stretching vibrations of phenolic compounds. After nanoparticle synthesis (KNP), this band shifted to 3240 cm^−1^ and decreased in transmittance intensity, indicating the involvement of hydroxyl-containing compounds in AgNP formation. Although FTIR alone cannot unequivocally distinguish between their reducing and surface-binding roles, phenolic hydroxyl groups may contribute to Ag^+^ reduction based on their known electron-donating properties and the decrease in antioxidant activity observed after nanoparticle synthesis.

**Figure 6:**
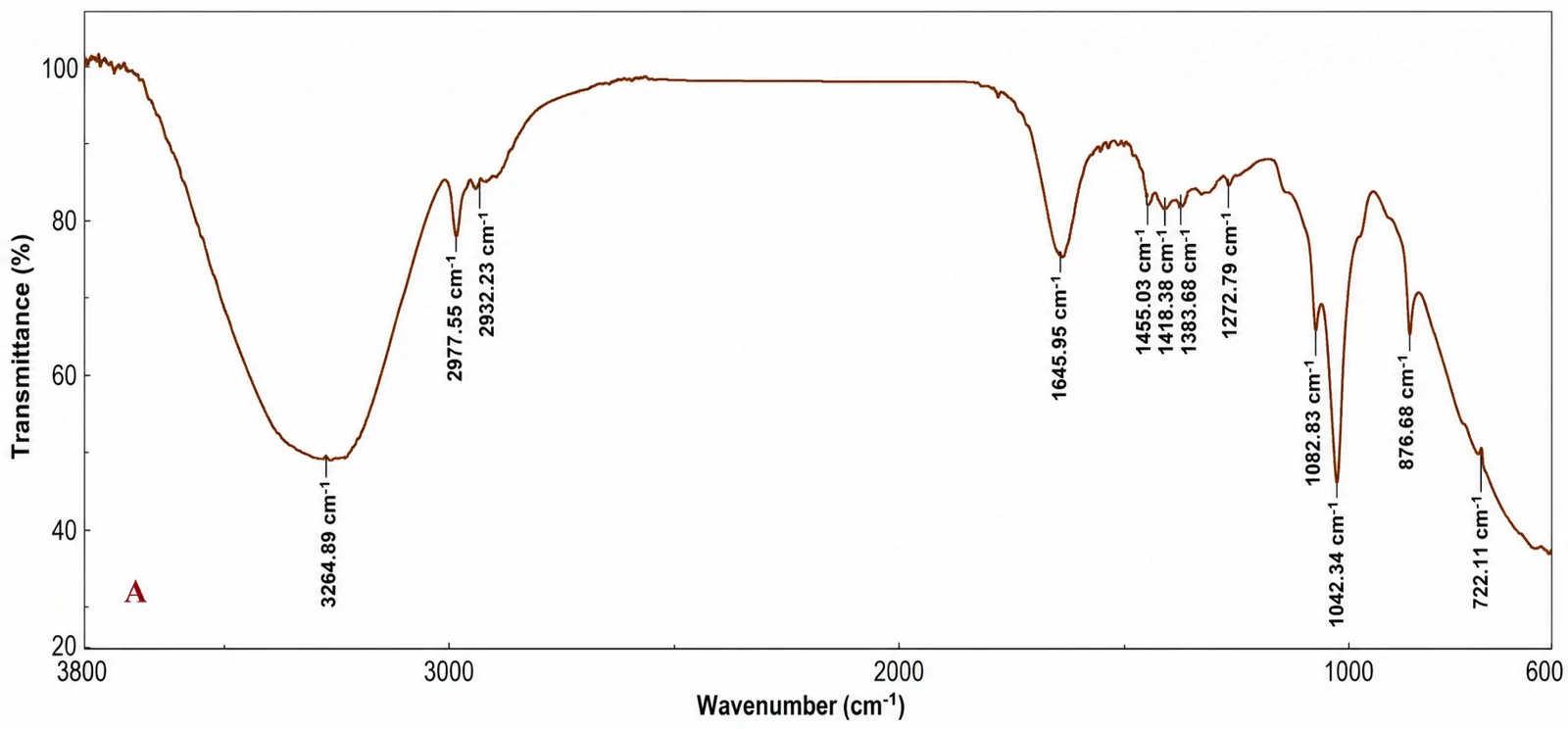

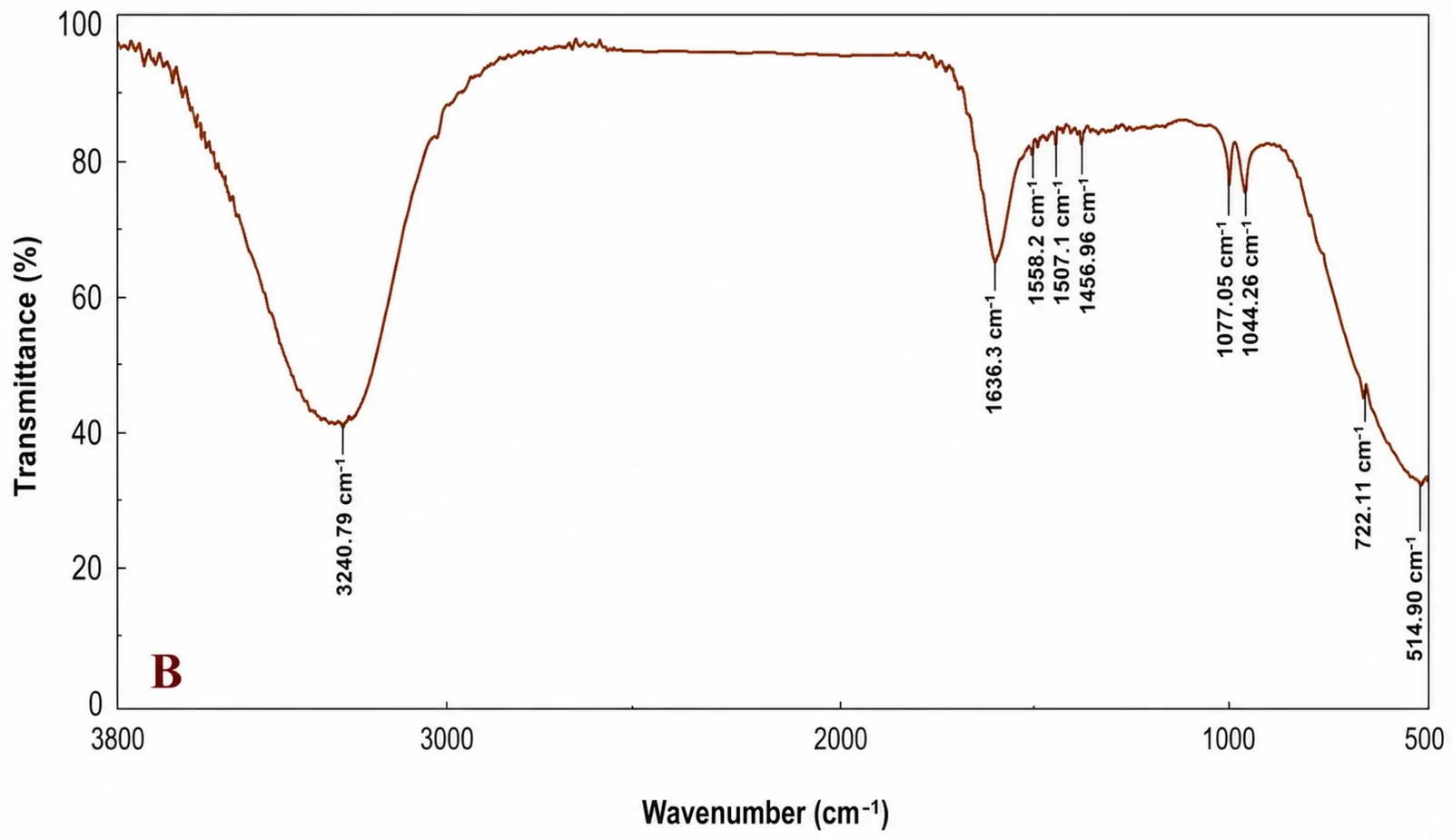
ATR-FTIR of the ethanolic extract of *Diospyros kaki* L. (**A**) and its AgNPs (**B**).

Similarly, the characteristic carbonyl stretching band shifted from 1645 cm^−1^ (K) to 1636 cm^−1^ (KNP), suggesting interaction between C=O-containing biomolecules and the silver nanoparticle surface. The shift toward lower wavenumbers is consistent with changes in the C=O bonding environment associated with surface interactions. Additional bands observed in the 1558–1507 cm^−1^ region in KNP may be attributed to amide II vibrations, suggesting that nitrogen-containing biomolecules could participate in interactions with the nanoparticle surface and potentially contribute to capping and stabilization. In the fingerprint region (1200–1000 cm^−1^), changes were detected in the C–O stretching bands (1082/1042 cm^−1^ in K vs. 1077/1044 cm^−1^ in KNP), further supporting the involvement of phenolic and carbohydrate-derived moieties in interactions with the nanoparticle surface. Figure 6 and Table 2 present the ATR-FTIR spectra and band assignments for the *Diospyros kaki* extract and the corresponding AgNP-containing sample. For the other samples, the spectra are presented in the Supplementary Material (Figures S1 and S2).

Comparable spectral modifications were observed for the extracts of *Citrus sinensis* and *Punica granatum* following silver nanoparticle synthesis (Table 2; Figures S1 and S2). For the orange extract (P), the characteristic absorption bands at 3326, 1647, and 1326 cm^−1^ shifted to 3250, 1636, and 1311 cm^−1^, corresponding to O–H stretching, C=O stretching, and C–N vibrations, respectively. Similarly, in the pomegranate extract (R), bands initially located at 3313, 1645, and 1330 cm^−1^ shifted to 3257, 1635, and 1342 cm^−1^ after nanoparticle formation. These spectral shifts are consistent with changes in the local bonding environment resulting from interactions between oxygen- and nitrogen-containing functional groups and the silver nanoparticle surface. Taken together with the antioxidant data, the changes in the O–H region are consistent with a potential reducing role of phenolic hydroxyl groups, whereas changes in the carbonyl, amide/C–N, and C–O regions provide evidence of interactions with the nanoparticle surface that may contribute to capping and stabilization. However, these assignments should be regarded as functional interpretations rather than an unequivocal distinction between reducing and stabilizing phytochemicals.

### 3.3. BF-STEM—EDS Analysis and Nanoparticle Size Distribution

**Figure 7:**
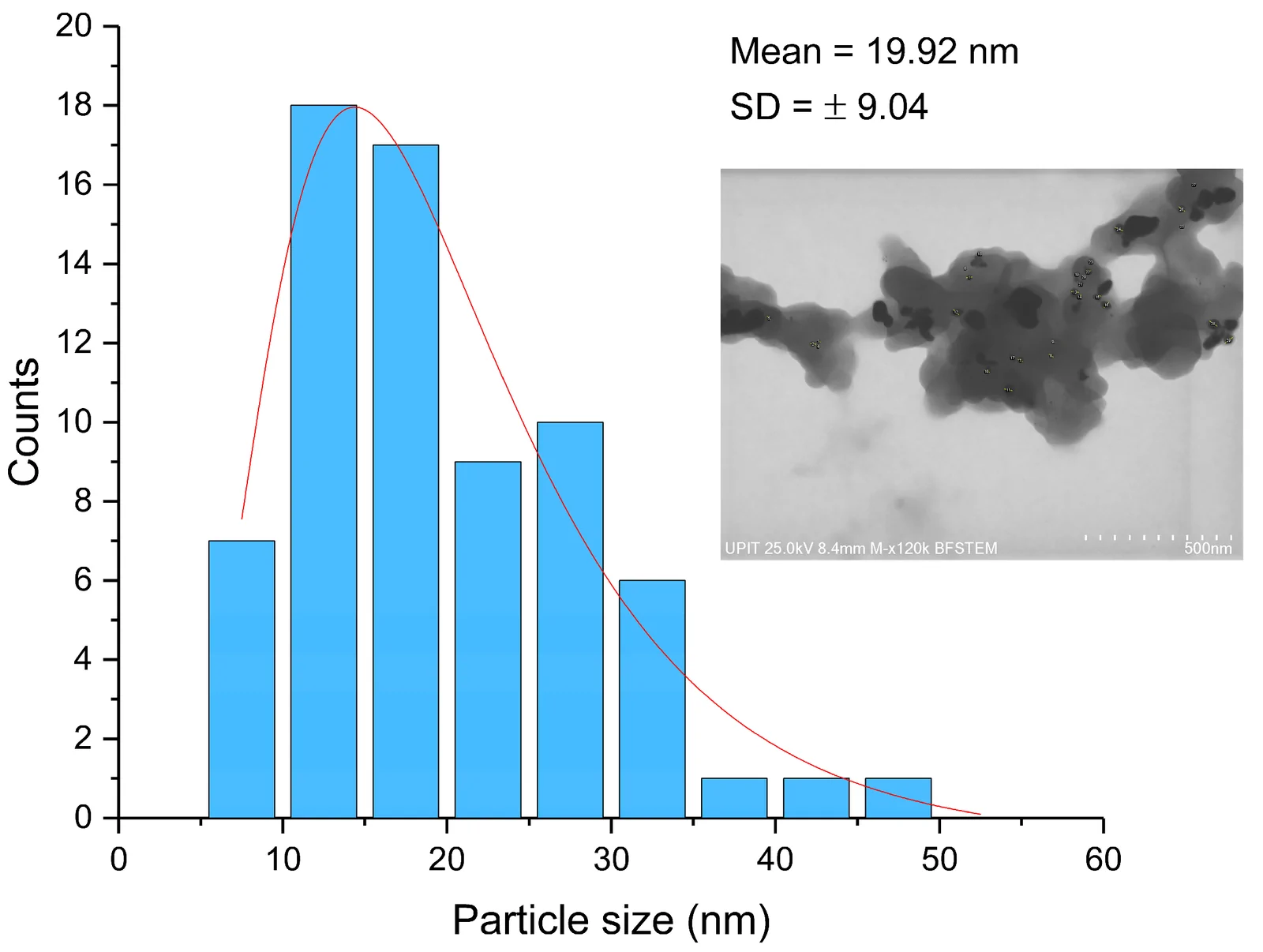
BF-STEM image and particle size distribution of AgNPs (KNP) synthesized using *Diospyros kaki* L. peel extract.

**Figure 8:**
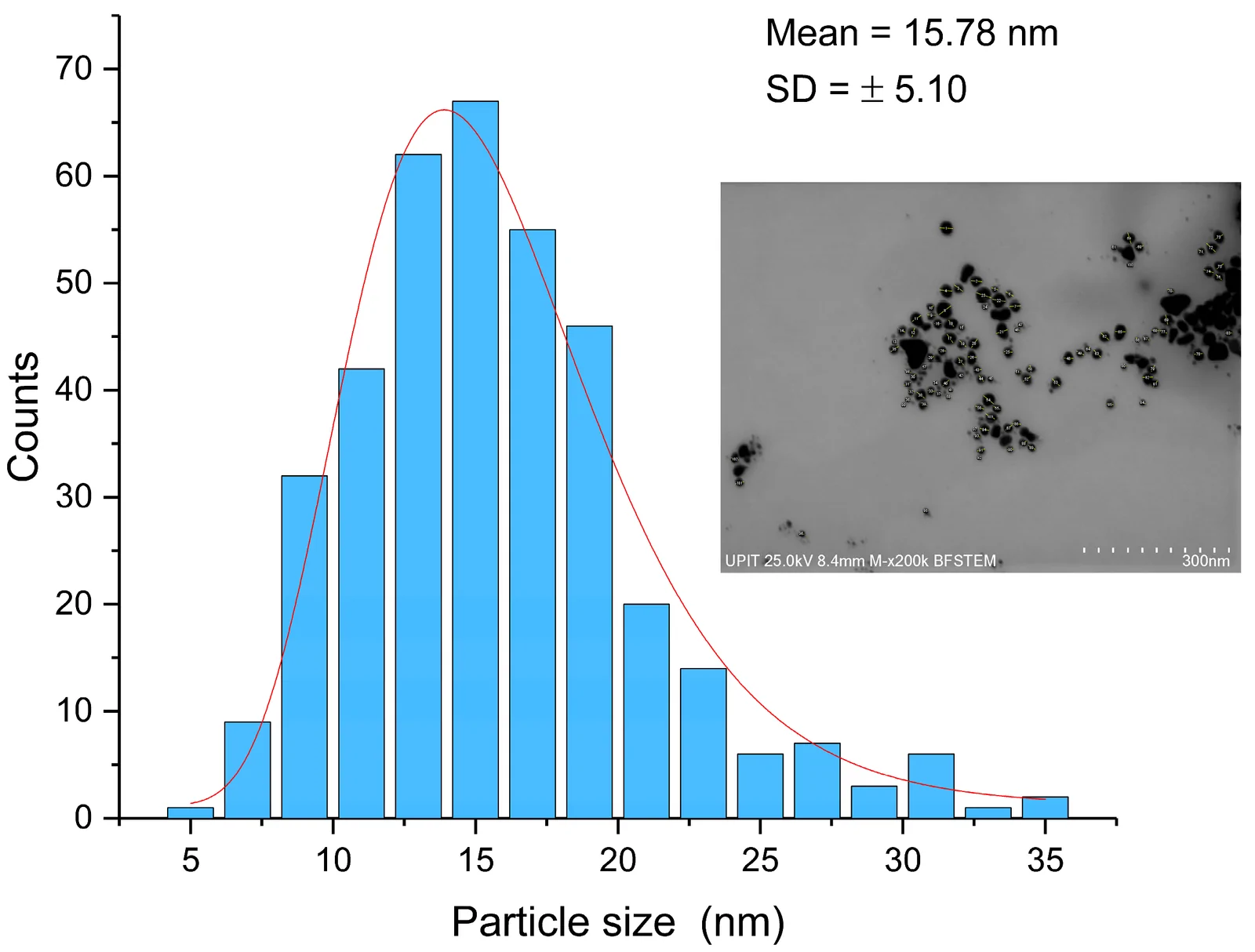
BF-STEM image and particle size distribution of AgNPs (PNP) synthesized using *Citrus sinensis* Osbeck peel extract.

**Figure 9:**
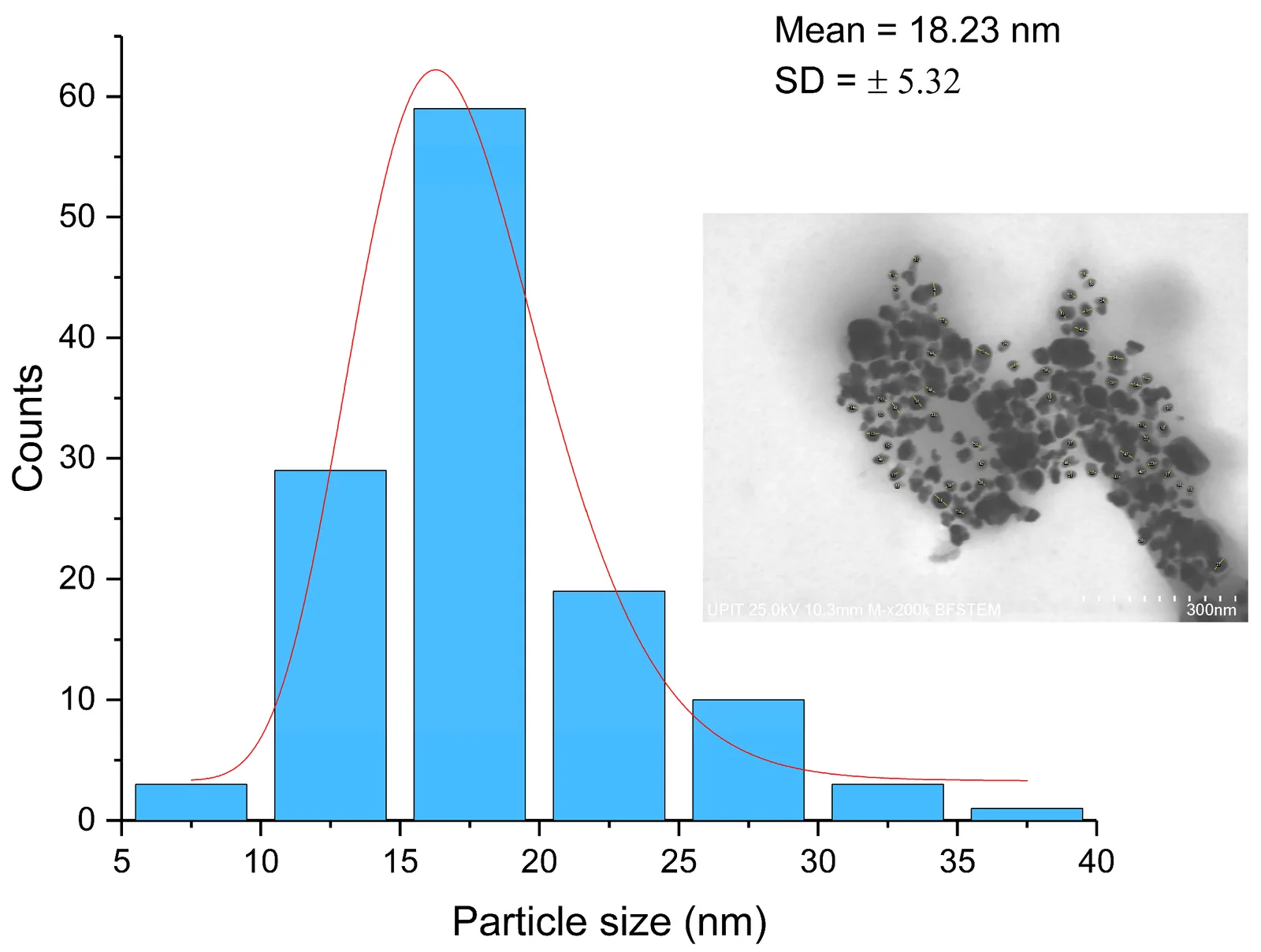
BF-STEM image and particle size distribution of AgNPs (RNP) synthesized using *Punica granatum* L. peel extract.

**Figure 10:**
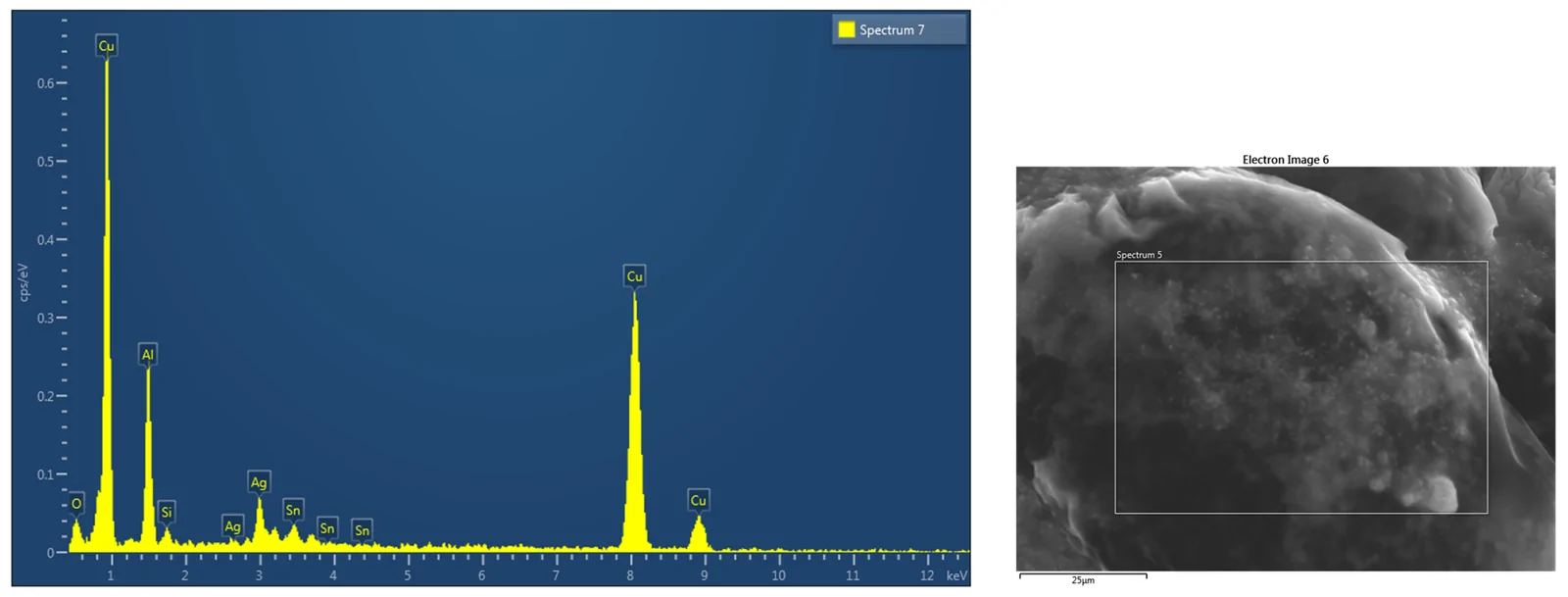
EDS spectra (**left**) of the scan area (**right**) obtained for the *Citrus sinensis* Osbeck extract (P) with AgNPs (PNP sample).

**Figure 11:**
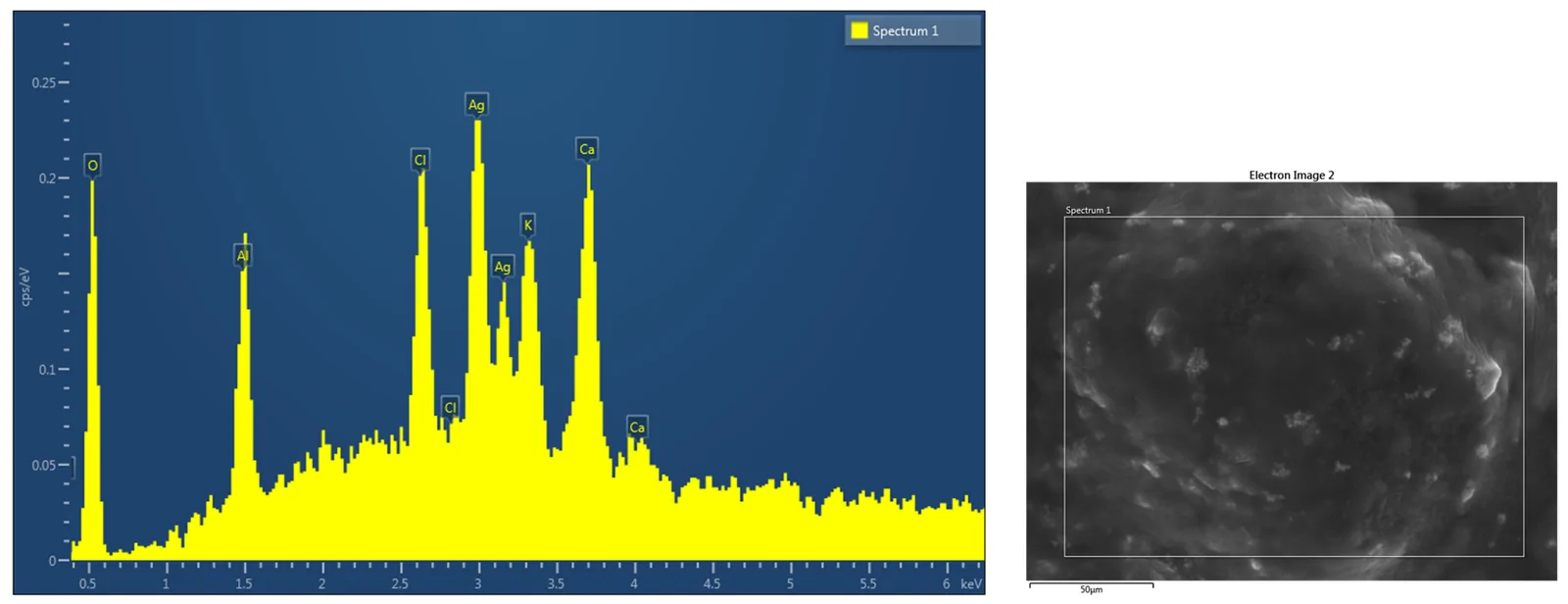
EDS spectra (**left**) of the scan area (**right**) obtained for the *Diospyros kaki* L. extract (K) with AgNPs (KNP sample).

**Figure 12:**
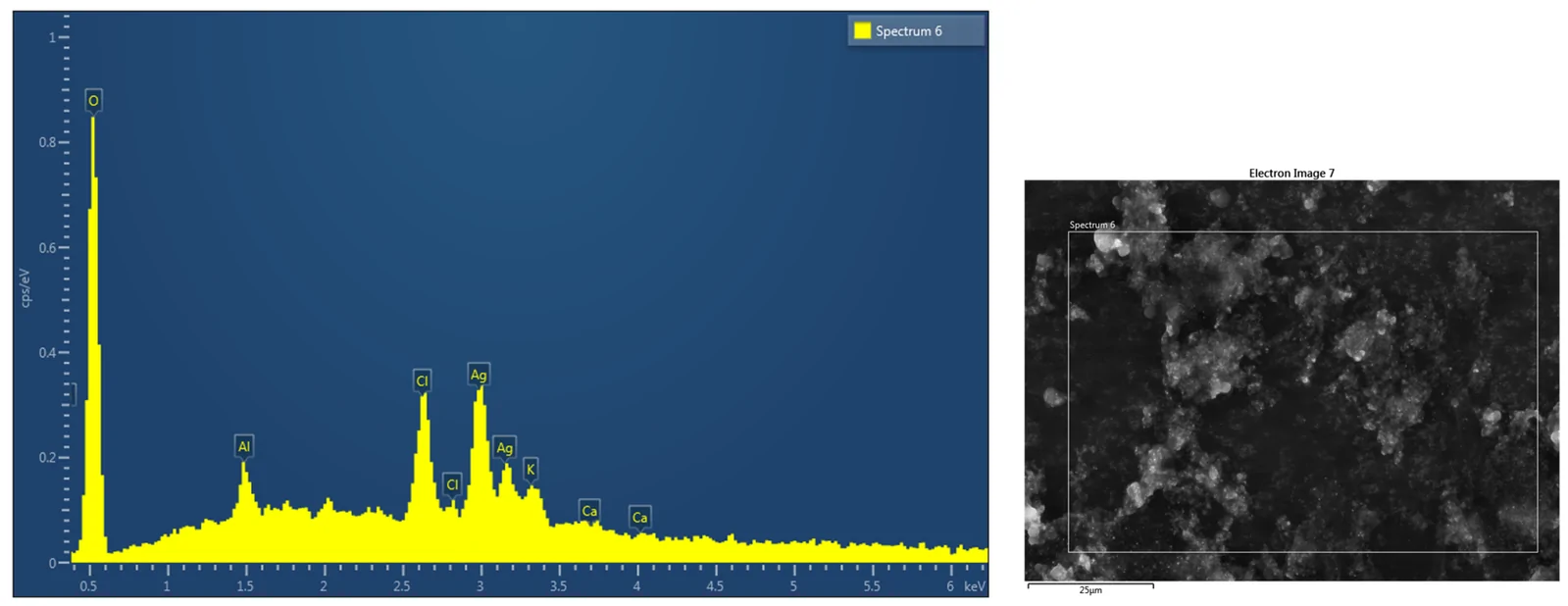
EDS spectra (**left**) of the scan area (**right**) obtained for the *Punica granatum* L. extract (R) with AgNPs (RNP sample).

The BF-STEM analysis revealed predominantly spherical AgNPs in all three phytosynthesis preparations. Particle size distribution analysis showed mean particle diameters of 19.92 ± 9.04 nm for KNP (*Diospyros kaki*), 15.78 ± 5.10 nm for PNP (*Citrus sinensis*), and 18.23 ± 5.32 nm for RNP (*Punica granatum*) (Figure 7, Figure 8 and Figure 9). The presence of characteristic chemical elements (O, Ca, K), as well as Ag originating from the AgNO_3_ solution and Cu and C from the grid, was highlighted in the EDS spectra (Figure 10, Figure 11 and Figure 12).

### 3.4. Antioxidant Activity of Extracts and AgNP-Containing Phytosynthesis Preparations

The DPPH assay is commonly used to determine the antioxidant capacity of extracts and AgNP mixtures. The antioxidant activity of the AgNP-containing phytosynthesis preparations was lower than that of the corresponding initial extracts (Table 3). The greatest reduction was observed in the orange peel extract (P), from 90.72% to 35.17%, in the sample in which a rapid and pronounced color change of the extract was observed after adding the silver nitrate solution (PNP) (Figure 1).

### 3.5. Influence of Extracts and AgNP-Containing Phytosynthesis Preparations on Growth, Biomass, Pigment, and Polyphenol Content in Triticum aestivum L.

**Figure 13:**
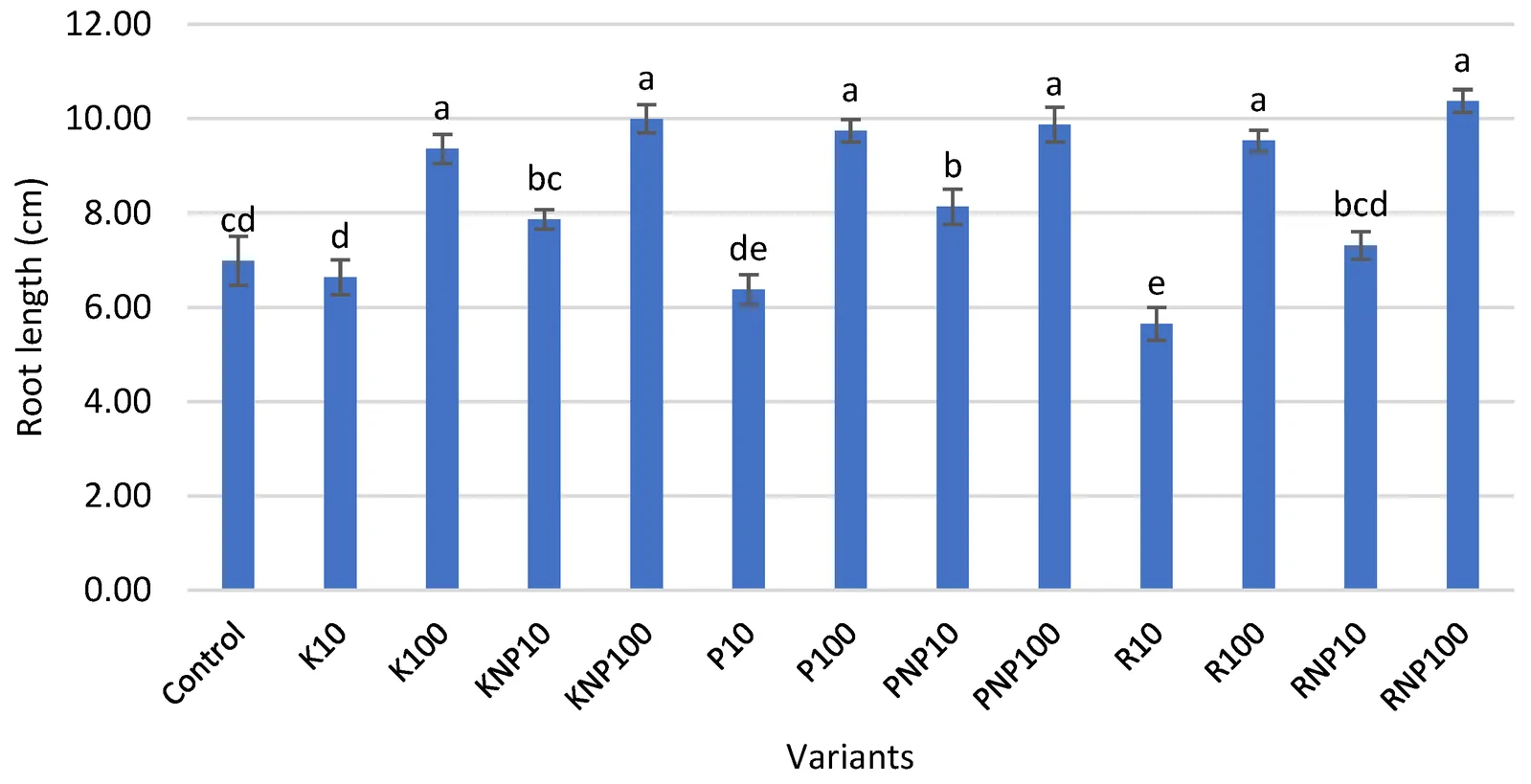
Influence of extracts and AgNP-containing phytosynthesis preparations obtained from the peel of *Punica granatum* L. (R), *Diospyros kaki* L. (K) and *Citrus sinensis* Osbeck (P) on root growth in *Triticum aestivum* L. (a–e, the interpretation of the significance of the differences by means of the Duncan test, *p* < 0.05).

**Figure 14:**
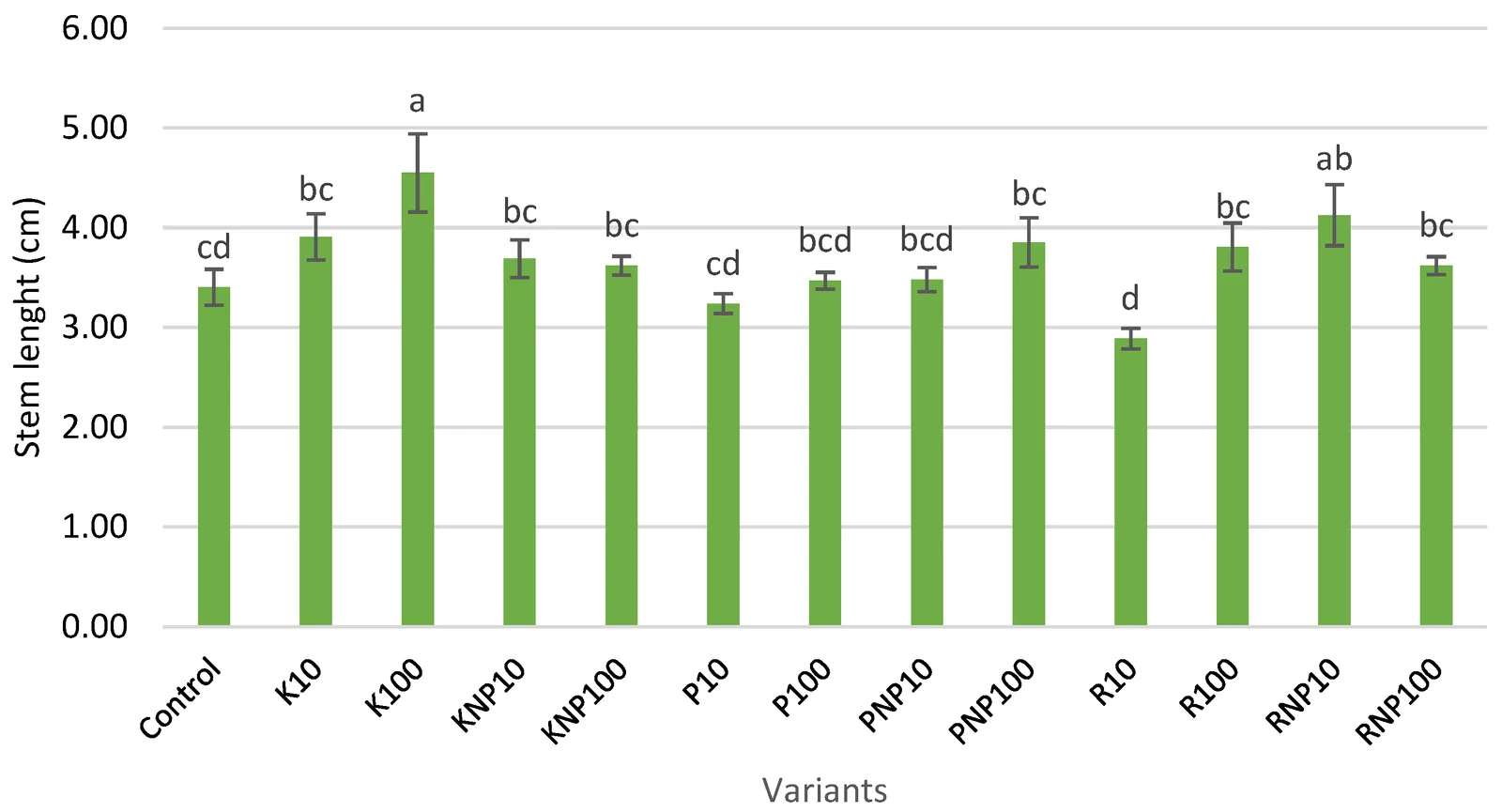
Influence of extracts and AgNP-containing phytosynthesis preparations obtained from the peel of *Punica granatum* L. (R), *Diospyros kaki* L. (K) and *Citrus sinensis* Osbeck (P) on stem growth in *Triticum aestivum* L. (a–d, the interpretation of the significance of the differences by means of the Duncan test, *p* < 0.05).

**Figure 15:**
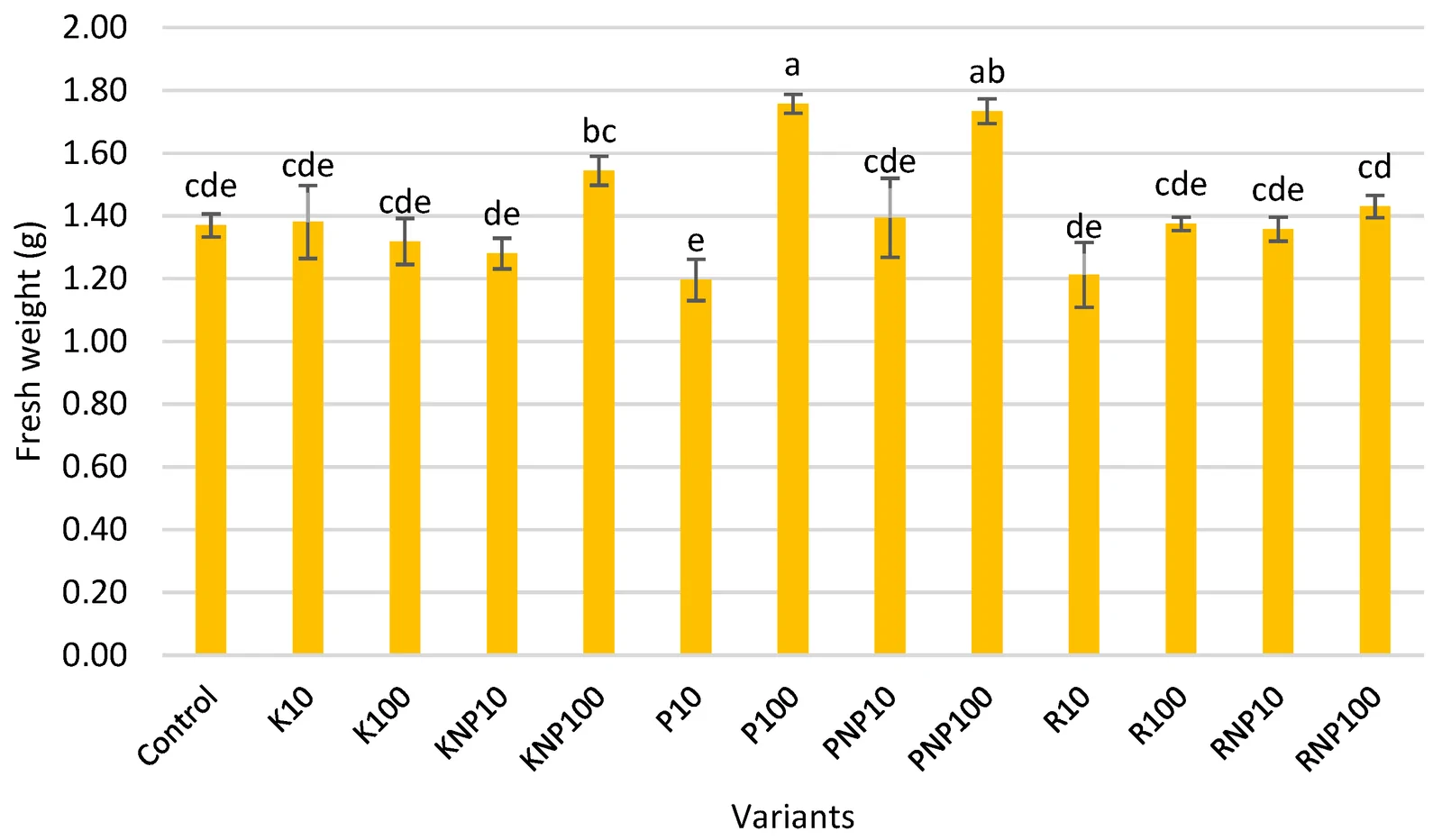
Influence of extracts and AgNP-containing phytosynthesis preparations obtained from the peel of *Punica granatum* L. (R), *Diospyros kaki* L. (K) and *Citrus sinensis* Osbeck (P) on fresh weight in *Triticum aestivum* L. (a–e, the interpretation of the significance of the differences by means of the Duncan test, *p* < 0.05).

The variants exposed to extracts and AgNP mixtures, diluted 100 times, significantly stimulated root growth in *T. aestivum*, compared to the control (Figure S3). Also, the 10-fold diluted AgNP-containing phytosynthesis preparations showed a positive influence on root growth compared with the corresponding extract-only variants (Figure 13). In the case of the R10 variant, a significant inhibition of the growth in length of both the root and the stem was observed (Figure 13 and Figure 14). A similar situation (growth inhibition) was also recorded for the P10 variant; thus, the two extract-only variants, R10 and P10, showed the lowest wet biomass values (Figure 15) because of the inhibition of root and stem growth. In the case of wet weight, the P100 extract and the corresponding PNP100 phytosynthesis preparation showed the highest biomass values, 1.76 and 1.73 g, respectively.

**Figure 16:**
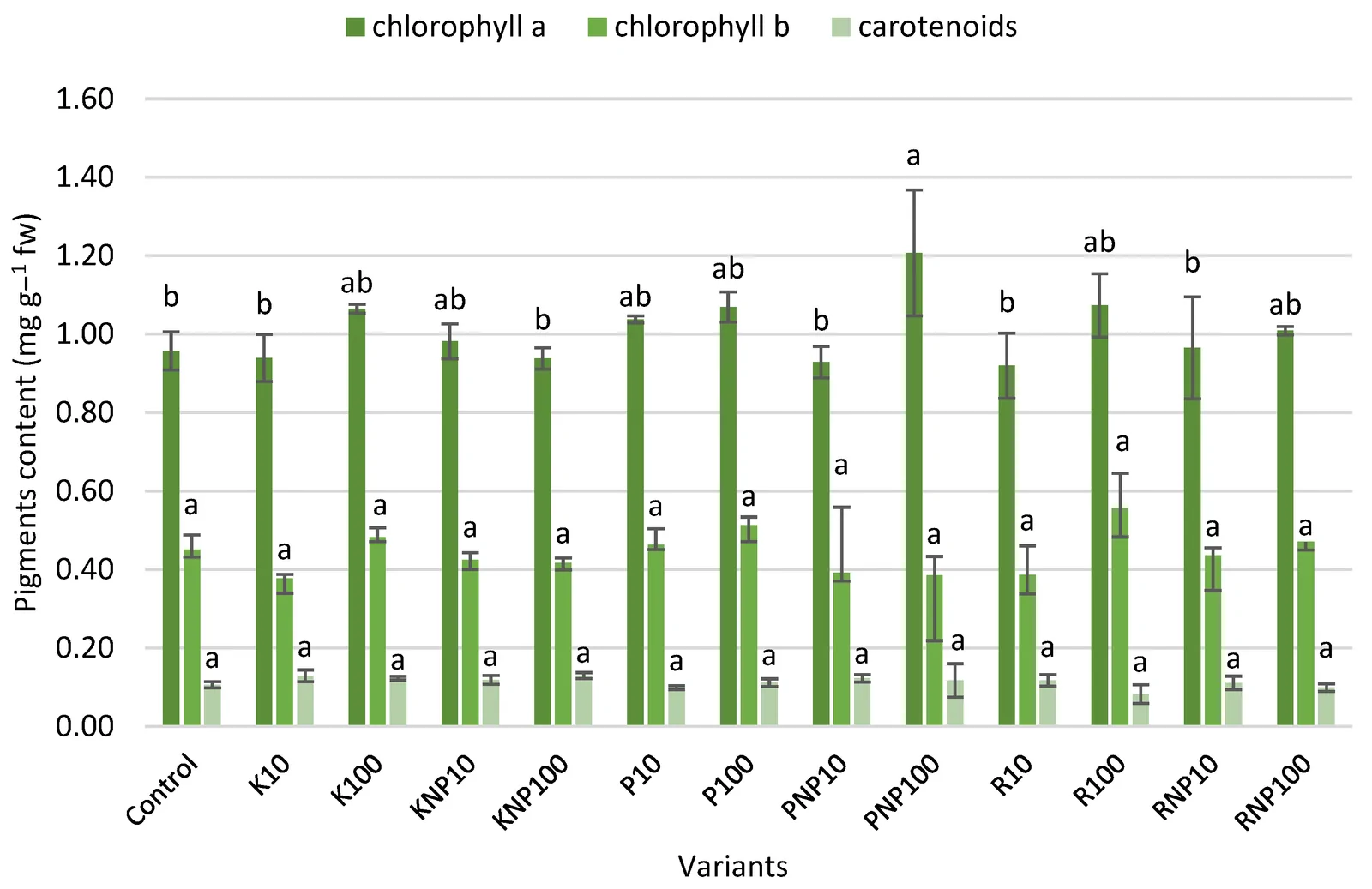
Influence of extracts and AgNP-containing phytosynthesis preparations obtained from the peel of *Punica granatum* L. (R), *Diospyros kaki* L. (K) and *Citrus sinensis* Osbeck (P) on pigment content in *Triticum aestivum* L. (a, b, the interpretation of the significance of the differences by means of the Duncan test, *p* < 0.05).

The highest chlorophyll concentration was determined in the PNP100 variant (1.21 mg g^−1^ fw) and differed significantly from that recorded in the control (Figure 16). The application of extracts without nanoparticles, diluted 100 times (K100, P100, R100), led to an increase in the content of chlorophyll *a* and *b* compared to the control. The values recorded for the content of carotenoids fell within the range [0.10; 0.14] mg g^−1^ fw; thus, there are no significant differences between the variants.

**Figure 17:**
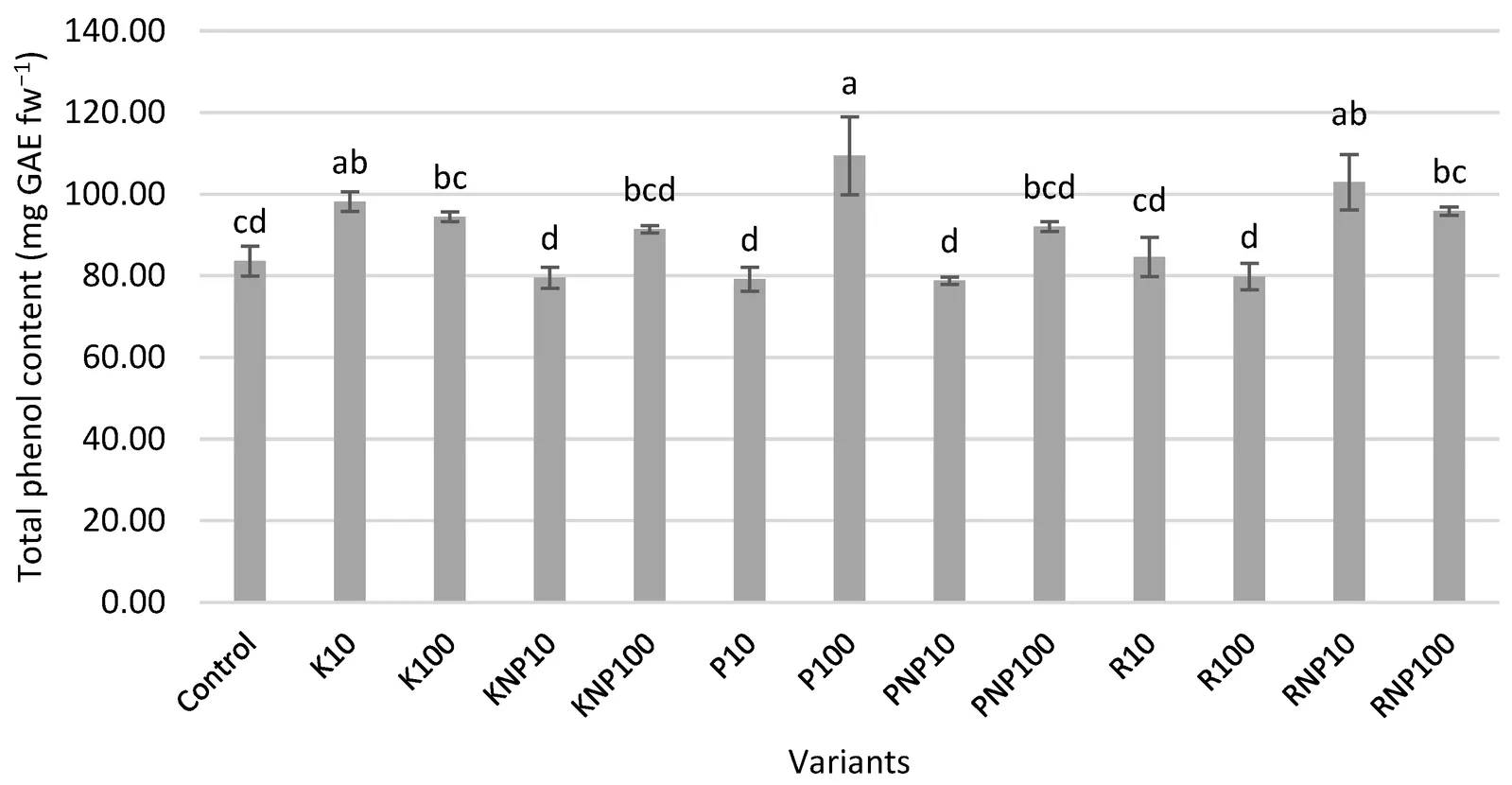
Influence of extracts and AgNP-containing phytosynthesis preparations obtained from the peel of *Punica granatum* L. (R), *Diospyros kaki* L. (K) and *Citrus sinensis* Osbeck (P) on total phenol content in *Triticum aestivum* L. (a–d, the interpretation of the significance of the differences by means of the Duncan test, *p* < 0.05).

The content of polyphenols in leaves increased, in general, compared to the control, because of exposure to the extracts, the maximum value (109 mg gallic acid equivalent—GAE g fw^−1^) being obtained for the P100 variant (Figure 17). Values of approximately 79 mg GAE were recorded for the KNP10, P10, PNP10 and R100 variants, these being lower than the value recorded for the control.

### 3.6. Influence of Extracts and AgNP-Containing Phytosynthesis Preparations on Growth, Biomass, Pigment, and Polyphenol Content in Zea mays L.

**Figure 18:**
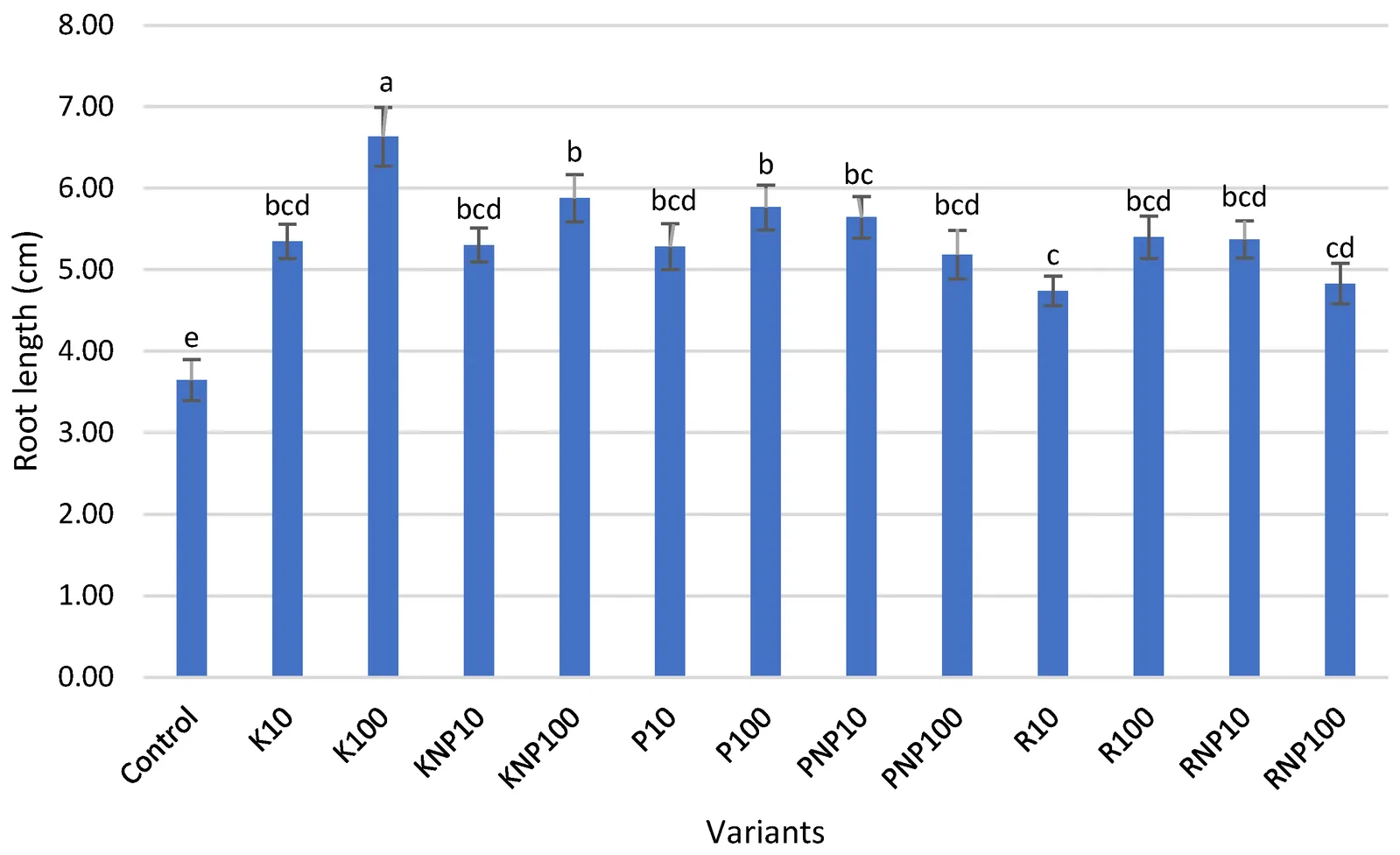
Influence of extracts and AgNP-containing phytosynthesis preparations obtained from the peel of *Punica granatum* L. (R), *Diospyros kaki* L. (K) and *Citrus sinensis* Osbeck (P) on root growth in *Zea mays* L. (a–e, the interpretation of the significance of the differences by means of the Duncan test, *p* < 0.05).

**Figure 19:**
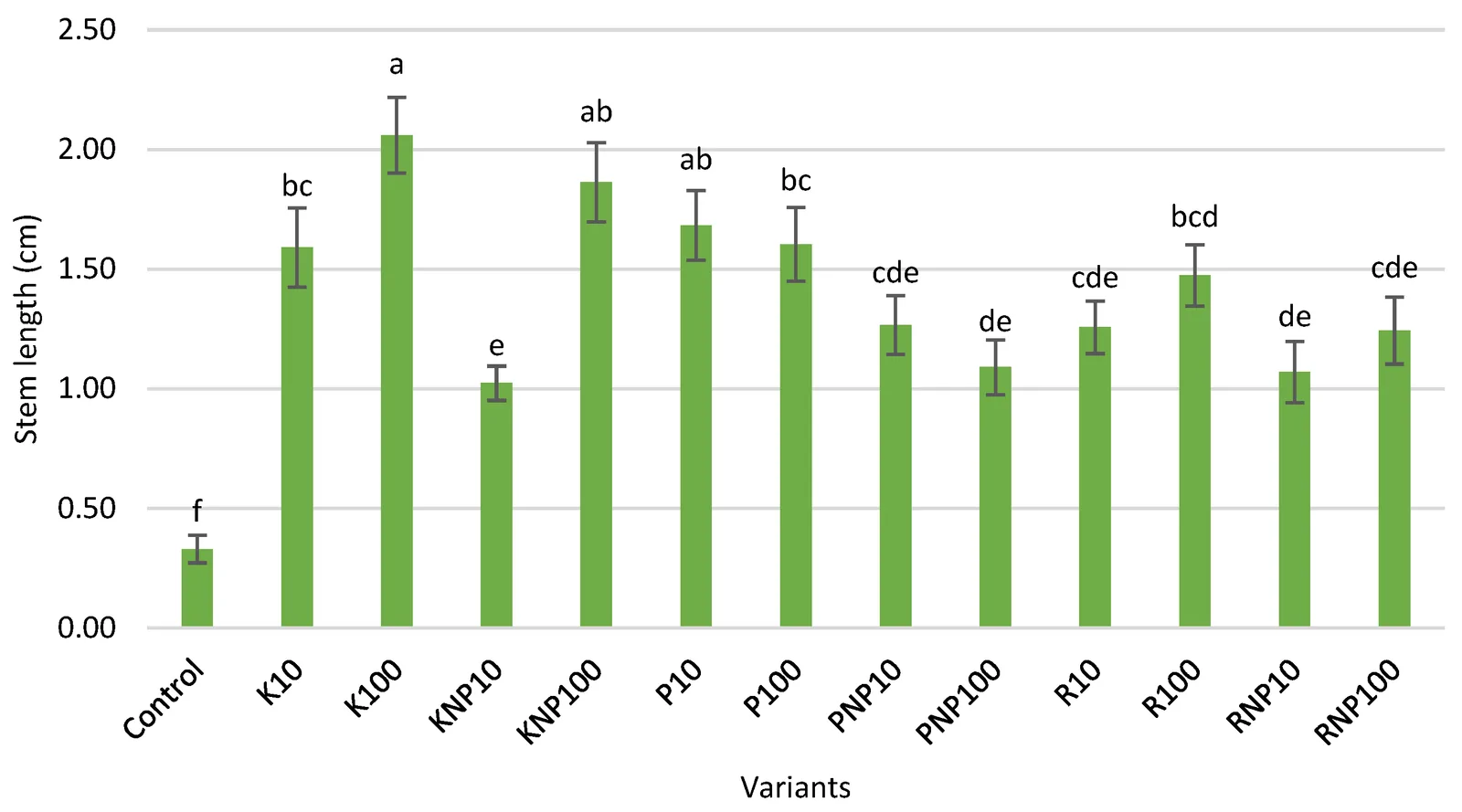
Influence of extracts and AgNP-containing phytosynthesis preparations obtained from the peel of *Punica granatum* L. (R), *Diospyros kaki* L. (K) and *Citrus sinensis* Osbeck (P) on stem growth in *Zea mays* L. (a–f, the interpretation of the significance of the differences by means of the Duncan test, *p* < 0.05).

**Figure 20:**
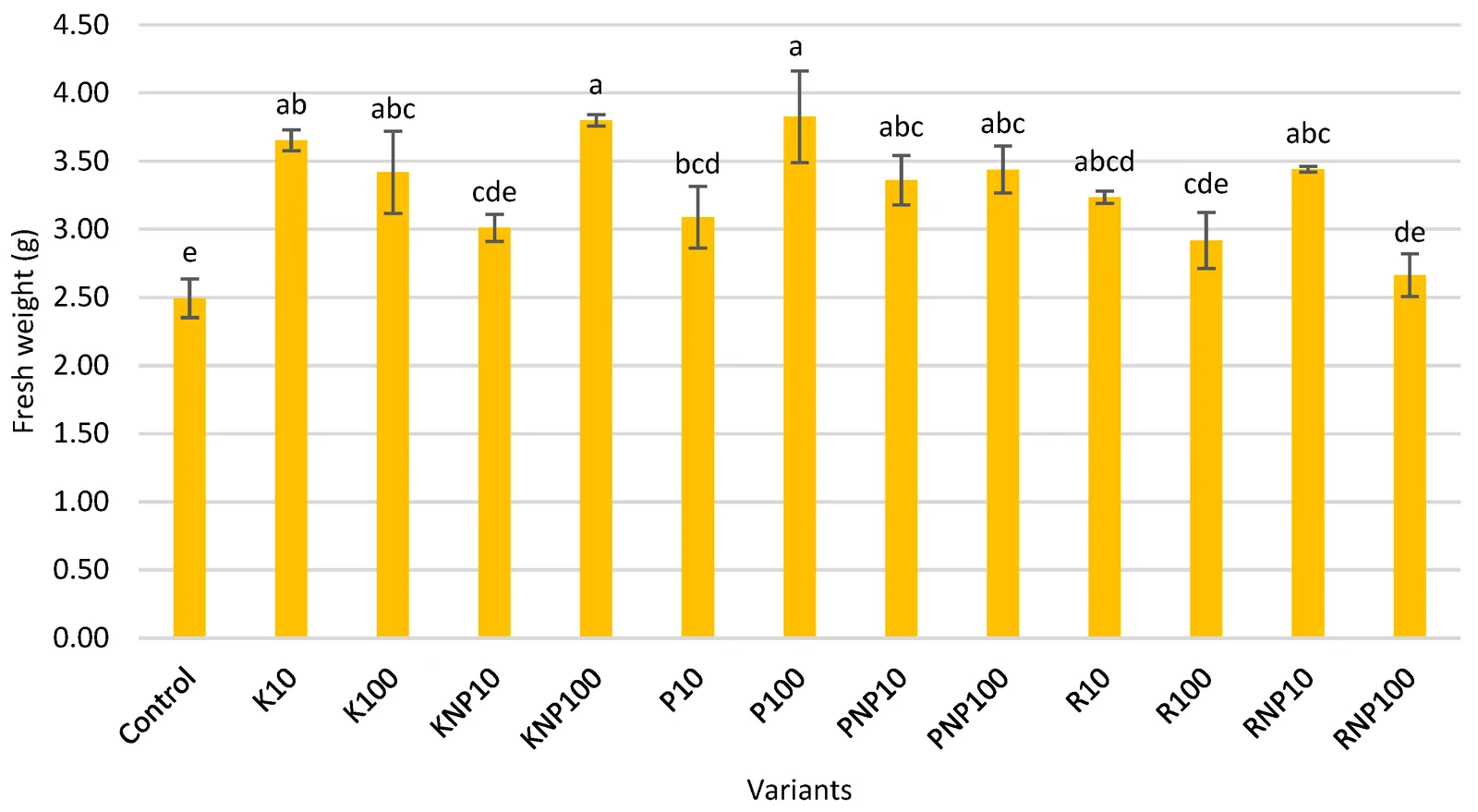
Influence of extracts and AgNP-containing phytosynthesis preparations obtained from the peel of *Punica granatum* L. (R), *Diospyros kaki* L. (K) and *Citrus sinensis* Osbeck (P) on fresh weight in *Zea mays* L. (a–e, the interpretation of the significance of the differences by means of the Duncan test, *p* < 0.05).

The growth of the root and stem in corn (Figure S4) was positively influenced after exposure of the caryopsis to the extracts with and without nanoparticles, regardless of the dilution (Figure 18 and Figure 19); therefore, the lowest value of the fresh weight (2.49 g) was recorded in the control (Figure 20). The highest values for the length of the root and stem were determined in the K100 variant, being approximately 2 and 4 times higher than those obtained in the control.

**Figure 21:**
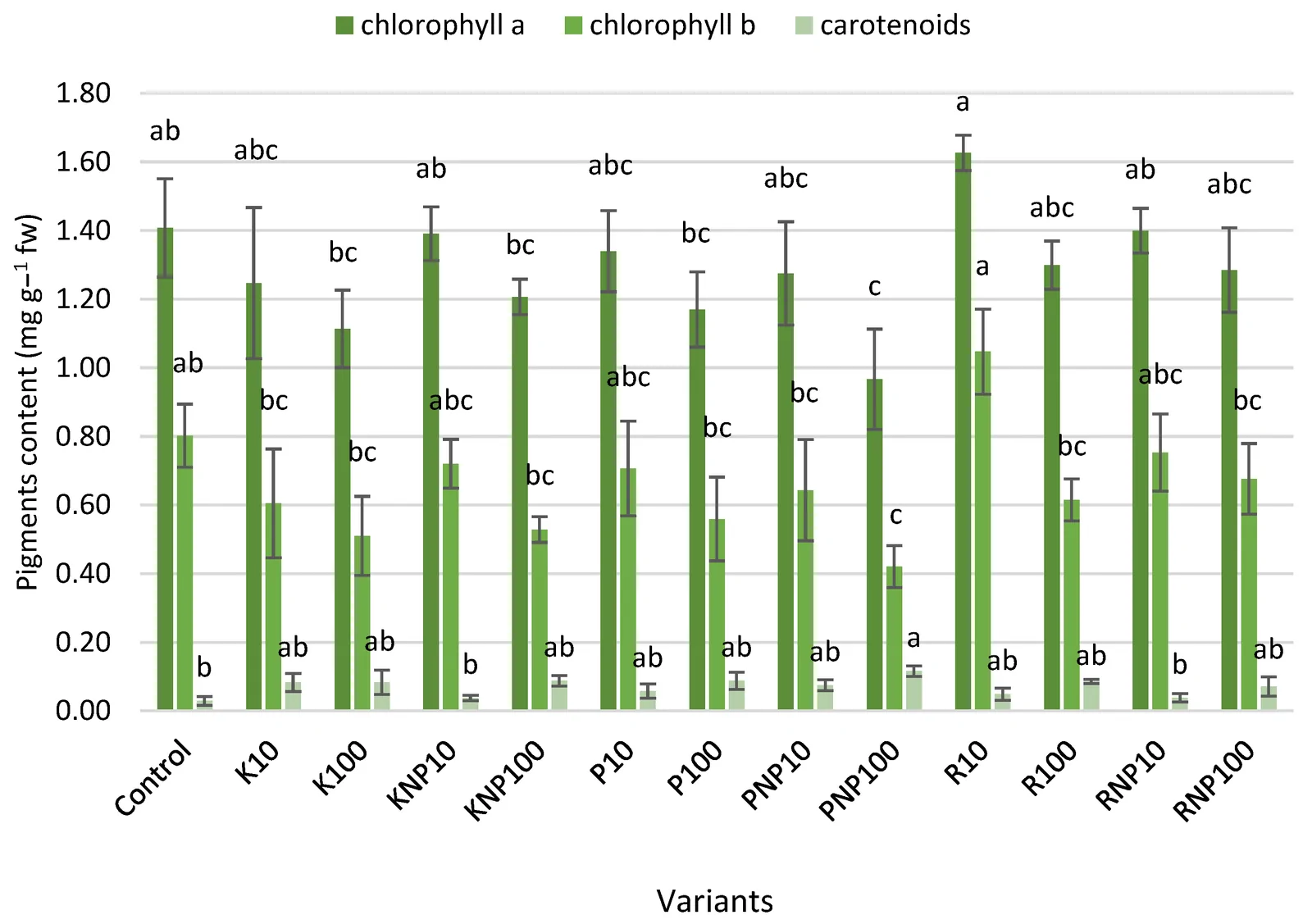
Influence of extracts and AgNP-containing phytosynthesis preparations obtained from the peel of *Punica granatum* L. (R), *Diospyros kaki* L. (K) and *Citrus sinensis* Osbeck (P) on pigment content in *Zea mays* L. (a–c, the interpretation of the significance of the differences by means of the Duncan test, *p* < 0.05).

In the case of the pigment content in the R10 and PNP100 variants, the maximum and minimum concentrations of chlorophyll *a* and *b* were recorded. The reduction in the amount of chlorophyll in the PNP100 variant is significant compared to the control, and the response of the plants to this stress factor consists of an increase in the concentration of carotenoid pigments that have a protective role (Figure 21).

**Figure 22:**
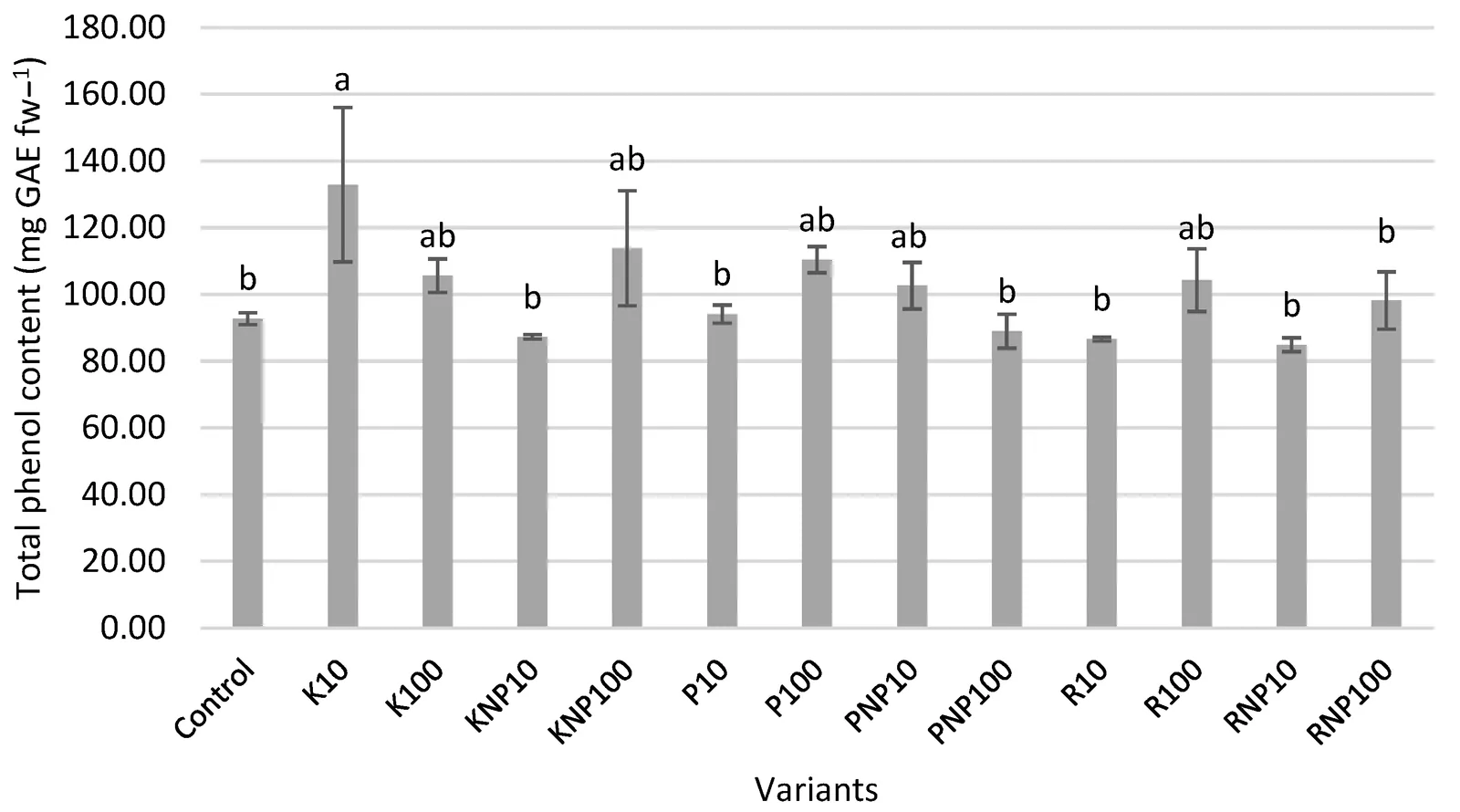
Influence of extracts and AgNP-containing phytosynthesis preparations obtained from the peel of *Punica granatum* L. (R), *Diospyros kaki* L. (K), and *Citrus sinensis* Osbeck (P) on total phenol content in *Zea mays* L. (a, b, the interpretation of the significance of the differences by means of the Duncan test, *p* < 0.05).

The determination of polyphenol content did not reveal significant differences between the control and the variants with extracts with and without nanoparticles, with one exception: the K10 variant, where the value of 132 mg GAE g fw^−1^ was recorded (Figure 22).

### 3.7. Influence of Extracts and AgNP-Containing Phytosynthesis Preparations on Growth, Biomass, Pigment, and Polyphenol Content in Cucumis sativus L.

**Figure 23:**
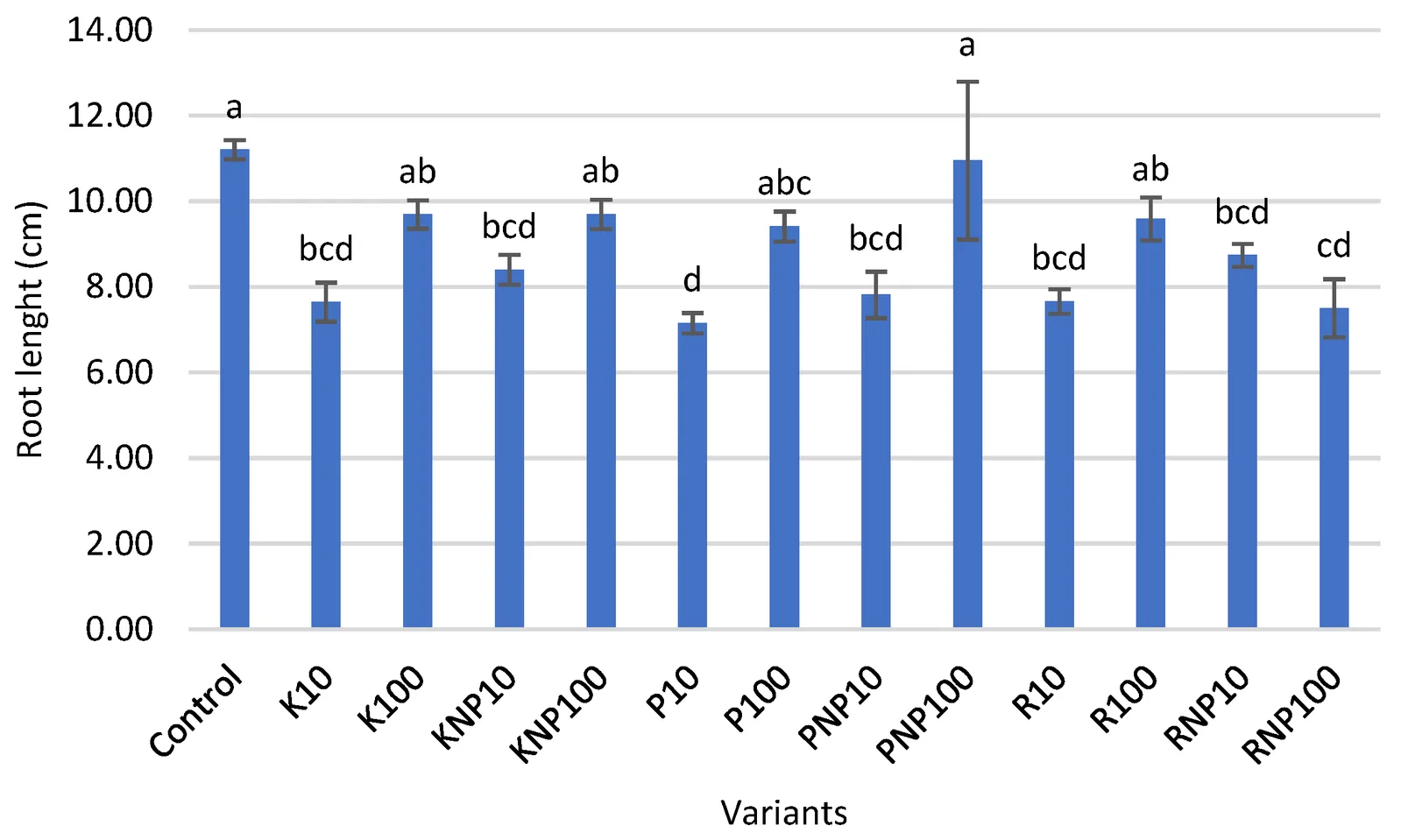
Influence of extracts and AgNP-containing phytosynthesis preparations obtained from the peel of *Punica granatum* L. (R), *Diospyros kaki* L. (K) and *Citrus sinensis* Osbeck (P) on root growth in *Cucumis sativus* L. (a–d, the interpretation of the significance of the differences by means of the Duncan test, *p* < 0.05).

**Figure 24:**
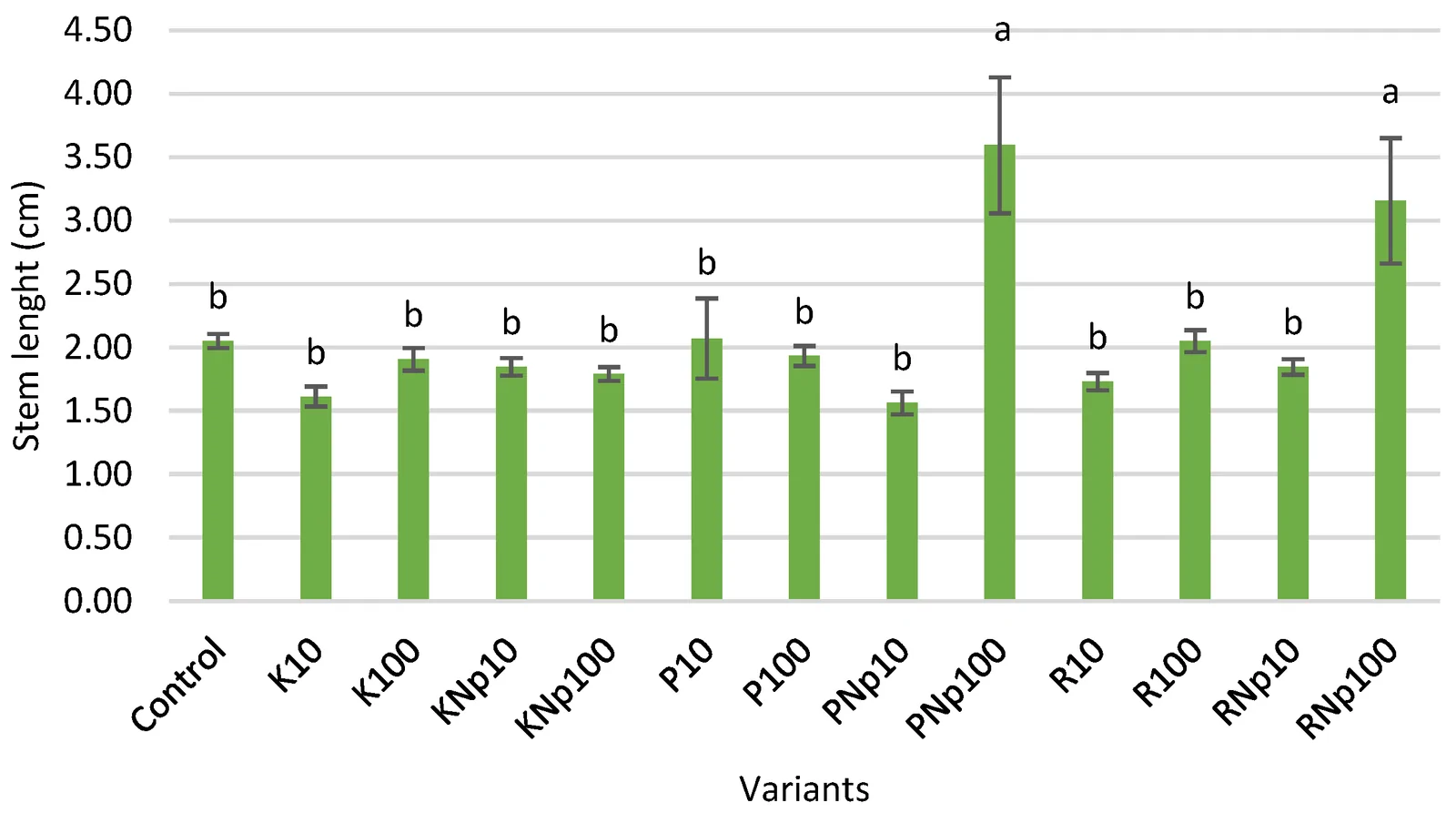
Influence of extracts and AgNP-containing phytosynthesis preparations obtained from the peel of *Punica granatum* L. (R), *Diospyros kaki* L. (K) and *Citrus sinensis* Osbeck (P) on stem growth in *Cucumis sativus* L. (a, b, the interpretation of the differences by means of the Duncan test, *p* < 0.05).

**Figure 25:**
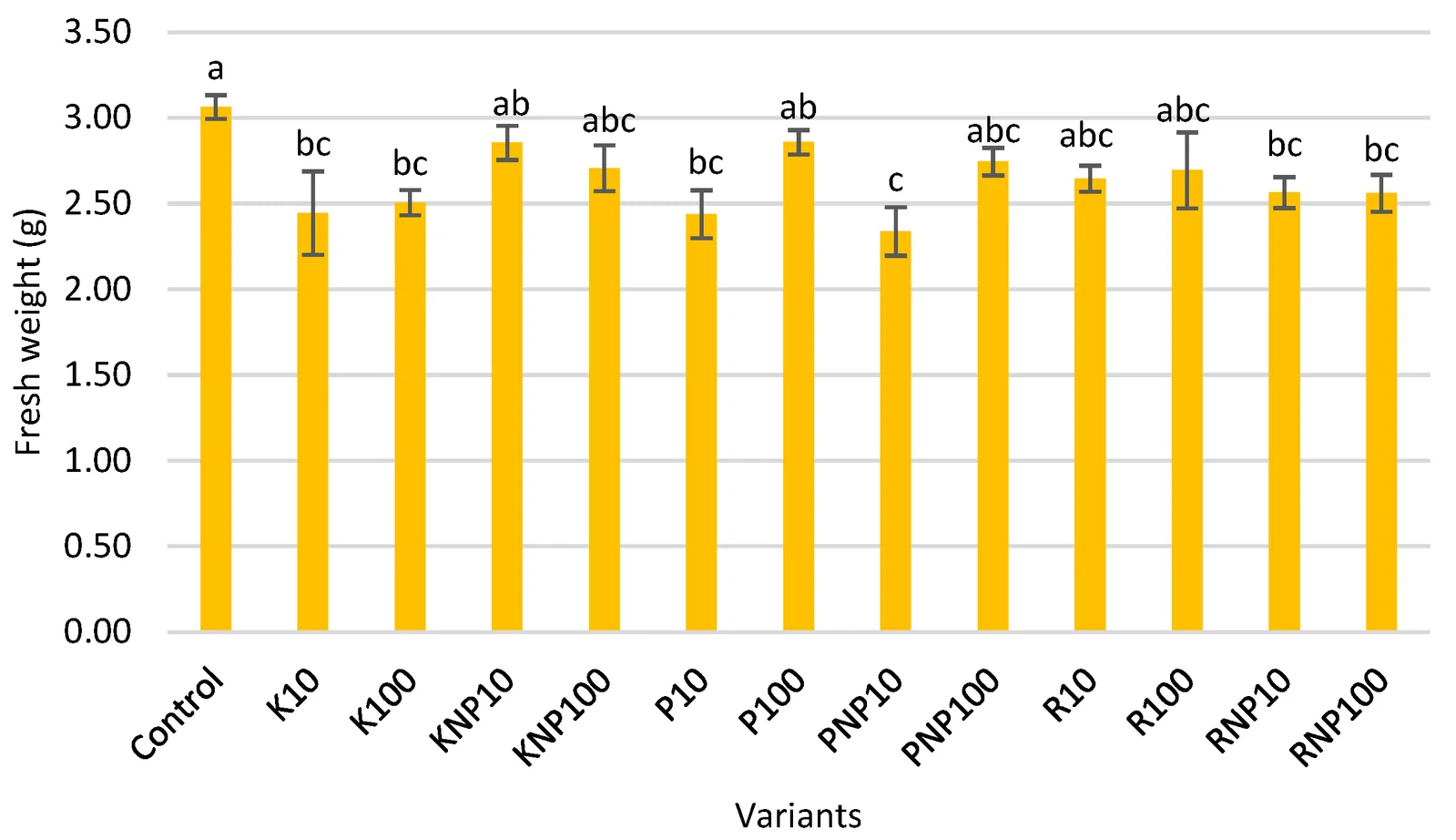
Influence of extracts and AgNP-containing phytosynthesis preparations obtained from the peel of *Punica granatum* L. (R), *Diospyros kaki* L. (K), and *Citrus sinensis* Osbeck (P) on stem growth in *Cucumis sativus* L. (a–c, the interpretation of the significance of the differences by means of the Duncan test, *p* < 0.05).

**Figure 26:**
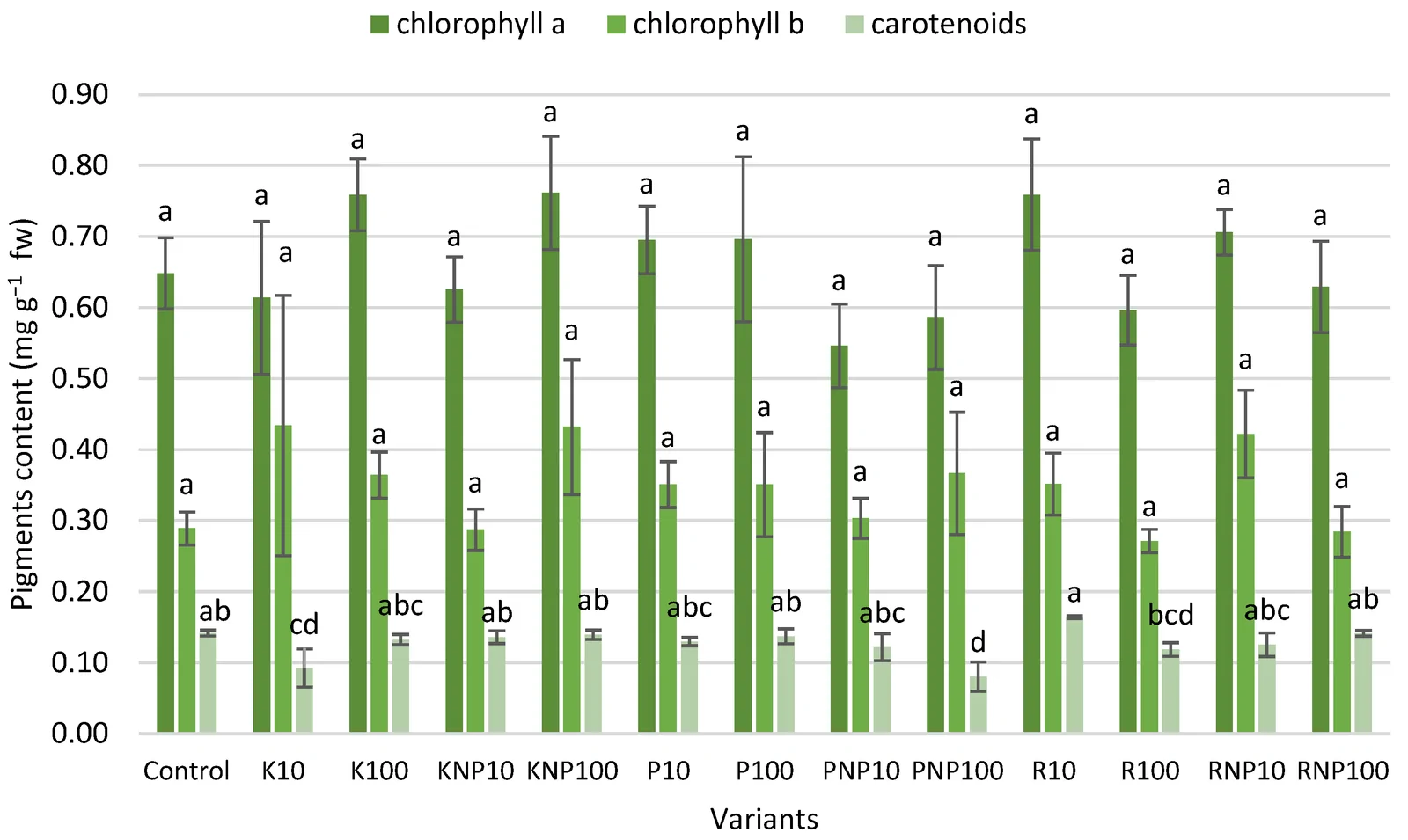
Influence of extracts and AgNP-containing phytosynthesis preparations obtained from the peel of *Punica granatum* L. (R), *Diospyros kaki* L. (K) and *Citrus sinensis* Osbeck (P) on pigment content in *Cucumis sativus* L. (a–d, the interpretation of the significance of the differences by means of the Duncan test, *p* < 0.05).

**Figure 27:**
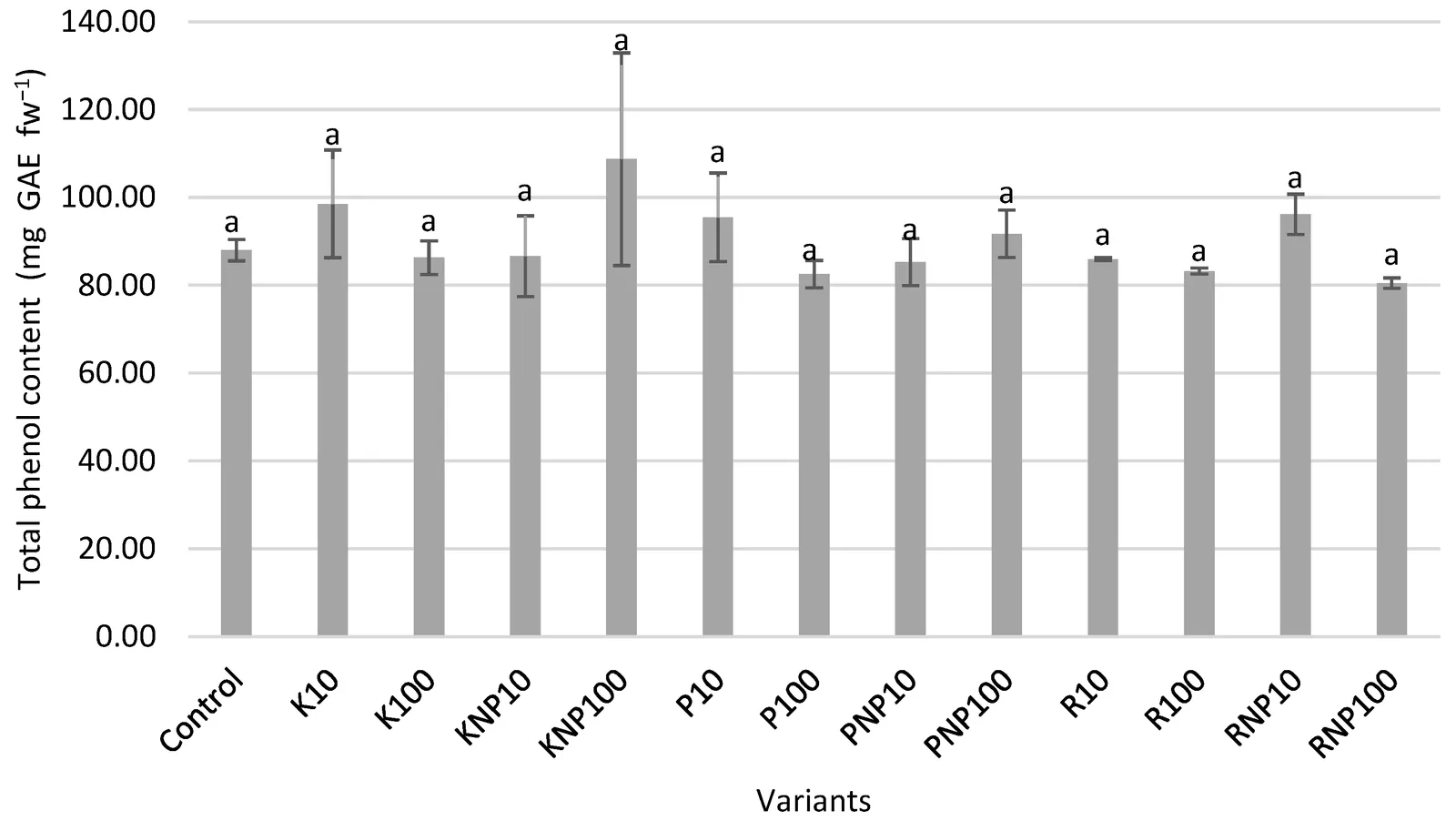
Influence of extracts and AgNP-containing phytosynthesis preparations obtained from the peel of *Punica granatum* L. (R), *Diospyros kaki* L. (K) and *Citrus sinensis* Osbeck (P) on total phenol content in *Cucumis sativus* L. (a, the interpretation of the significance of the differences by means of the Duncan test, *p* < 0.05).

Cucumber root growth was inhibited because of seed immersion in extracts with and without nanoparticles (Figure S5), the inhibition being significant in the case of variants diluted 10 times with and without nanoparticles (Figure 23). The values recorded for the stem lengths of cucumber seedlings fell within the interval [1.56; 2.05], with two exceptions: in the variants treated with orange and pomegranate extracts with nanoparticles diluted 100 times, the stem lengths were over 3 cm (Figure 24). As a result of the inhibition of root and stem growth, in the experimental variants with extracts, the value of wet biomass was lower than that determined in the control (3.06 g) (Figure 25). For chlorophyll *a* in variants K100, KNP100, P10, P100, R10, and RNP10, values of over 0.70 mg g^−1^ fw were obtained, which indicate a stimulation of the assimilatory pigment content compared to the control. In most variants treated with extracts, a tendency for chlorophyll *b* content to increase was observed compared to the control. The average values for the carotenoid content were 13 ± 1 mg g^−1^ fw (Figure 26). In the variants treated with persimmon and orange extracts, diluted 10 times, and in those with nanoparticles, diluted 100 times, the polyphenol content increased compared to the control, but the increases are not significant (Figure 27).

### 3.8. Comparative Antifungal Screening of Extracts and AgNP-Containing Phytosynthesis Preparations

The results obtained for the four fungal strains were synthesized in a heat map (Table 4) to clearly highlight the differences in sensitivity to the tested plant extracts and silver nanoparticles. In the case of the *Fusarium oxysporum* strain, the rapid and extensive mycelial development on the PDA medium did not allow the delimitation of clear and reproducible inhibition zones. For this reason, the values were not included in the comparative quantitative analysis, but the observed behavior suggests a higher tolerance of this species to the applied treatments. For the other strains, the heat map revealed a species-dependent antifungal response. The largest diameters of the inhibition zones were observed for *Penicillium hirsutum*, especially in the case of ethanolic extracts from pomegranate and orange peels. *Aspergillus niger* showed a more variable sensitivity, and *Botrytis cinerea* responded selectively to the treatments obtained from orange peels. Values represent mean inhibition zone diameters (mm).

### 3.9. Activity Against Penicillium hirsutum ATCC 52323

**Figure 28:**
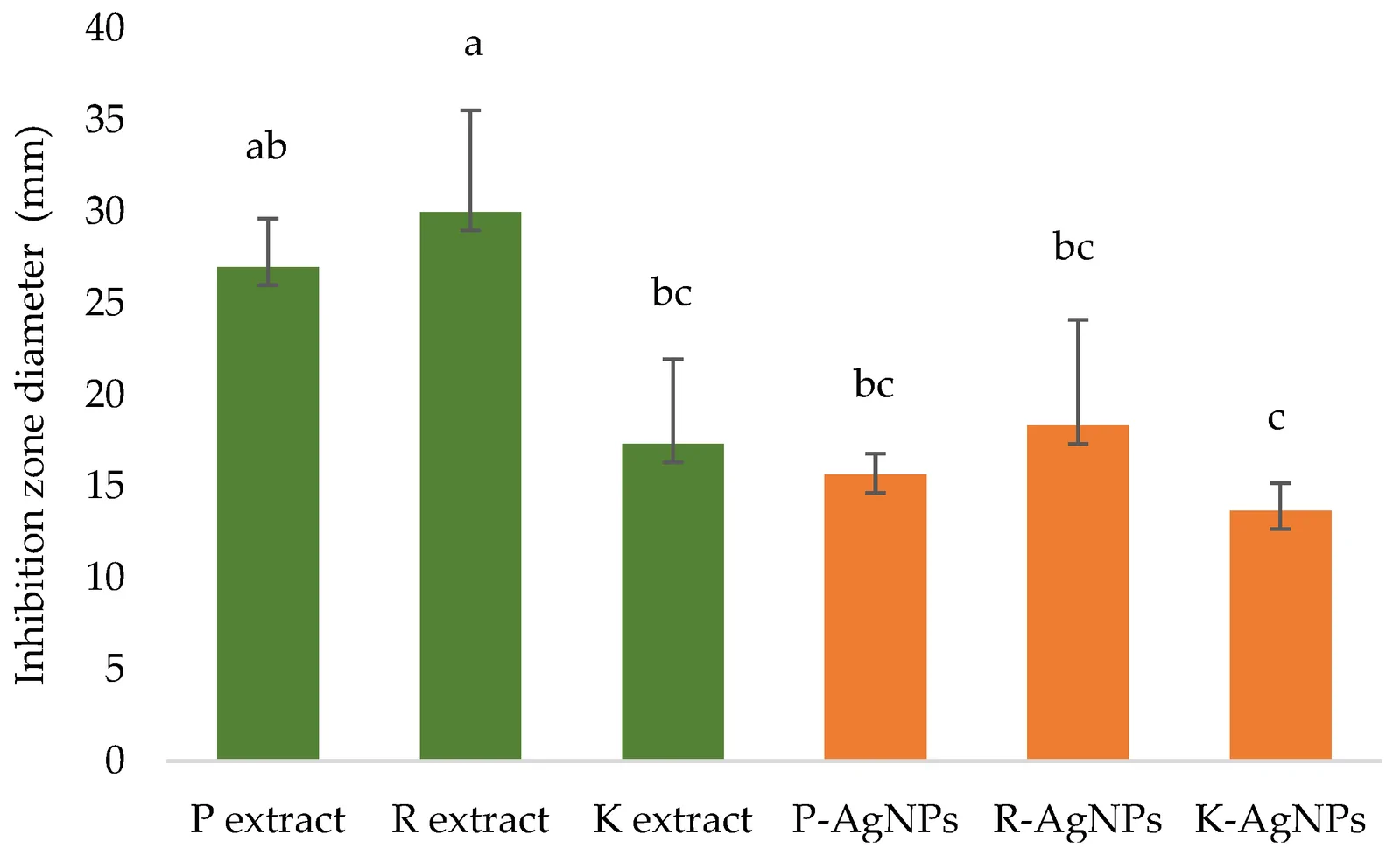
The average diameter of inhibition zones produced by plant extracts and the corresponding AgNP-containing phytosynthesis preparations against the *Penicillium hirsutum* ATCC 52323 strain. Error bars represent the standard deviation (*n* = 3), and different letters indicate statistically significant differences (Tukey HSD, *p* < 0.05).

Among the fungal strains investigated, *Penicillium hirsutum* showed the highest sensitivity to plant extracts and silver nanoparticles obtained by phytosynthesis. The diameters of the inhibition zones ranged between 13.67 and 30.00 mm, highlighting important differences between the analyzed treatments. The highest values were recorded for ethanolic extracts obtained from pomegranate and orange peels; the AgNP-containing phytosynthesis preparations generally showed lower antifungal effects. To evaluate the statistical significance of the differences observed between treatments, the data were analyzed by one-way ANOVA, followed by the post hoc Tukey HSD test (Figure 28). The results revealed the existence of significant differences between the samples (*p* < 0.05), confirming the influence of the type of extract and the phytosynthesis process on the antifungal activity against *P. hirsutum*.

The results obtained for *Penicillium hirsutum* revealed significant differences between the investigated treatments (ANOVA, *p* < 0.05). The largest zone of inhibition was recorded for the extract obtained from pomegranate peels, which presented superior antifungal activity compared to most of the analyzed samples. The orange peel extract also generated high values of the diameter of the zone of inhibition, without significant differences compared to the pomegranate extract. The values obtained for the persimmon peel extract and for the AgNP-containing preparations obtained using orange and pomegranate extracts were statistically comparable. The lowest activity was observed for the AgNP-containing preparation obtained using persimmon extract, which presented the smallest diameters of the zones of inhibition. Overall, the results indicate that the ethanolic extracts exerted a more pronounced antifungal effect against the *Penicillium hirsutum* strain than the corresponding AgNP-containing phytosynthesis preparations. This observation may reflect differences in the composition and availability of bioactive compounds between the crude extracts and the corresponding phytosynthesis preparations.

### 3.10. Activity Against Aspergillus niger ATCC 15475

**Figure 29:**
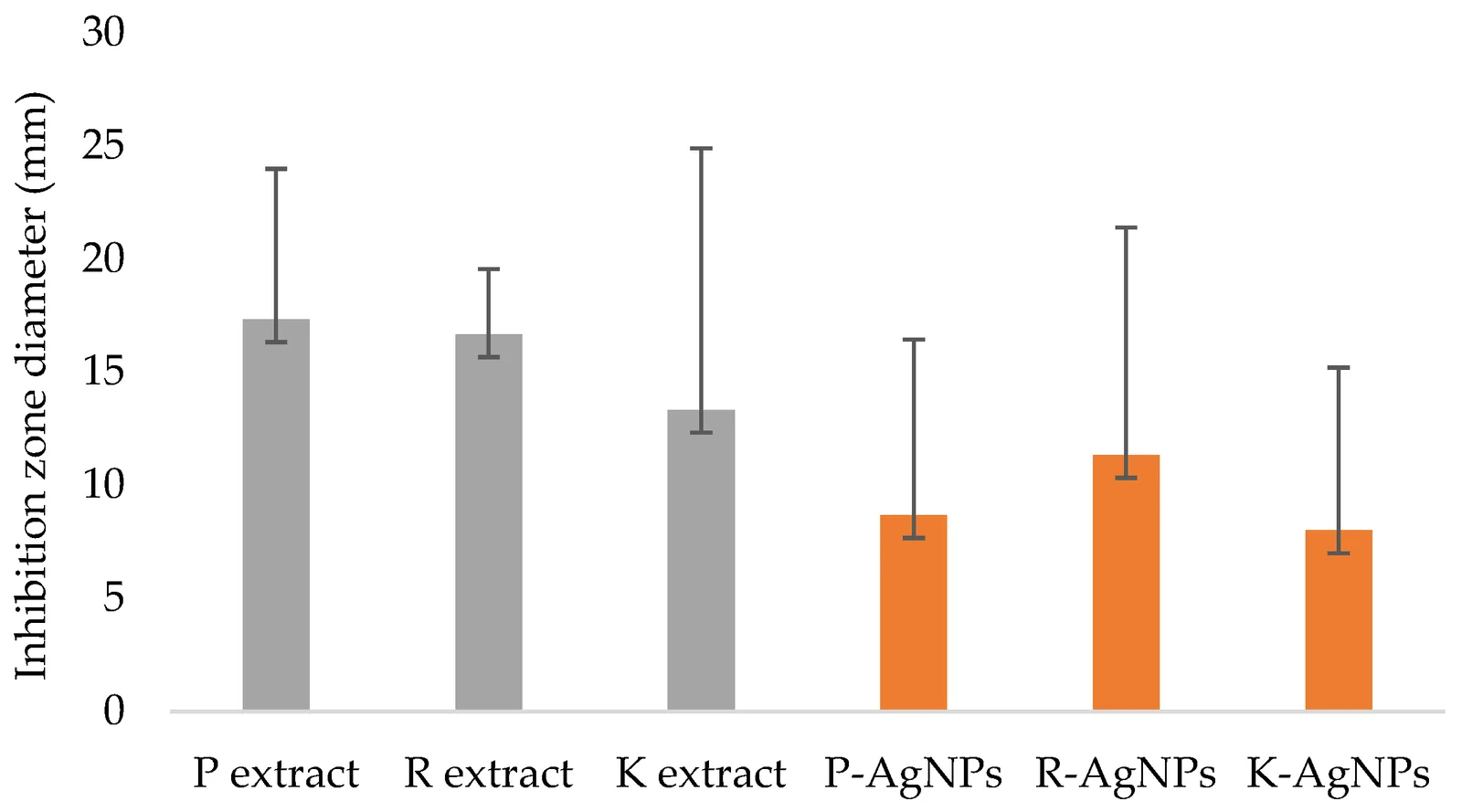
Average diameter of inhibition zones produced by plant extracts and the corresponding AgNP-containing phytosynthesis preparations against *Aspergillus niger* strain ATCC 15475. Error bars represent standard deviation (*n* = 3).

In contrast to the results obtained for *Penicillium hirsutum*, the strain *Aspergillus niger* showed a lower and more variable sensitivity to the investigated treatments (Figure 29). The diameters of the inhibition zones ranged between 8.00 and 17.33 mm, indicating the existence of numerical differences between the samples. However, the statistical analysis by one-way ANOVA did not reveal significant differences between the treatments (*p* > 0.05), suggesting that the observed variations cannot be attributed with certainty to the effect of the type of extract or the silver nanoparticles used.

The highest values of the diameter of the inhibition zone were recorded for the ethanolic extracts of orange and pomegranate peels, while the AgNP-containing phytosynthesis preparations generally showed comparable or slightly lower effects. However, the differences between the treatments remained within the limits of experimental variability. Although the ethanolic extract of orange peels presented the highest mean value of the diameter of the inhibition zone, statistical analysis did not reveal significant differences between treatments (ANOVA, *p* > 0.05). The relatively high variability observed for some samples, reflected by the high values of the standard deviation, contributed to the overlapping of the variation ranges and limited the evidence of statistical differences between treatments. These results suggest that *Aspergillus niger* exhibits a higher tolerance to the components present in the investigated extracts and AgNP-containing phytosynthesis preparations.

### 3.11. Activity Against Botrytis cinerea ATCC 11542

**Figure 30:**
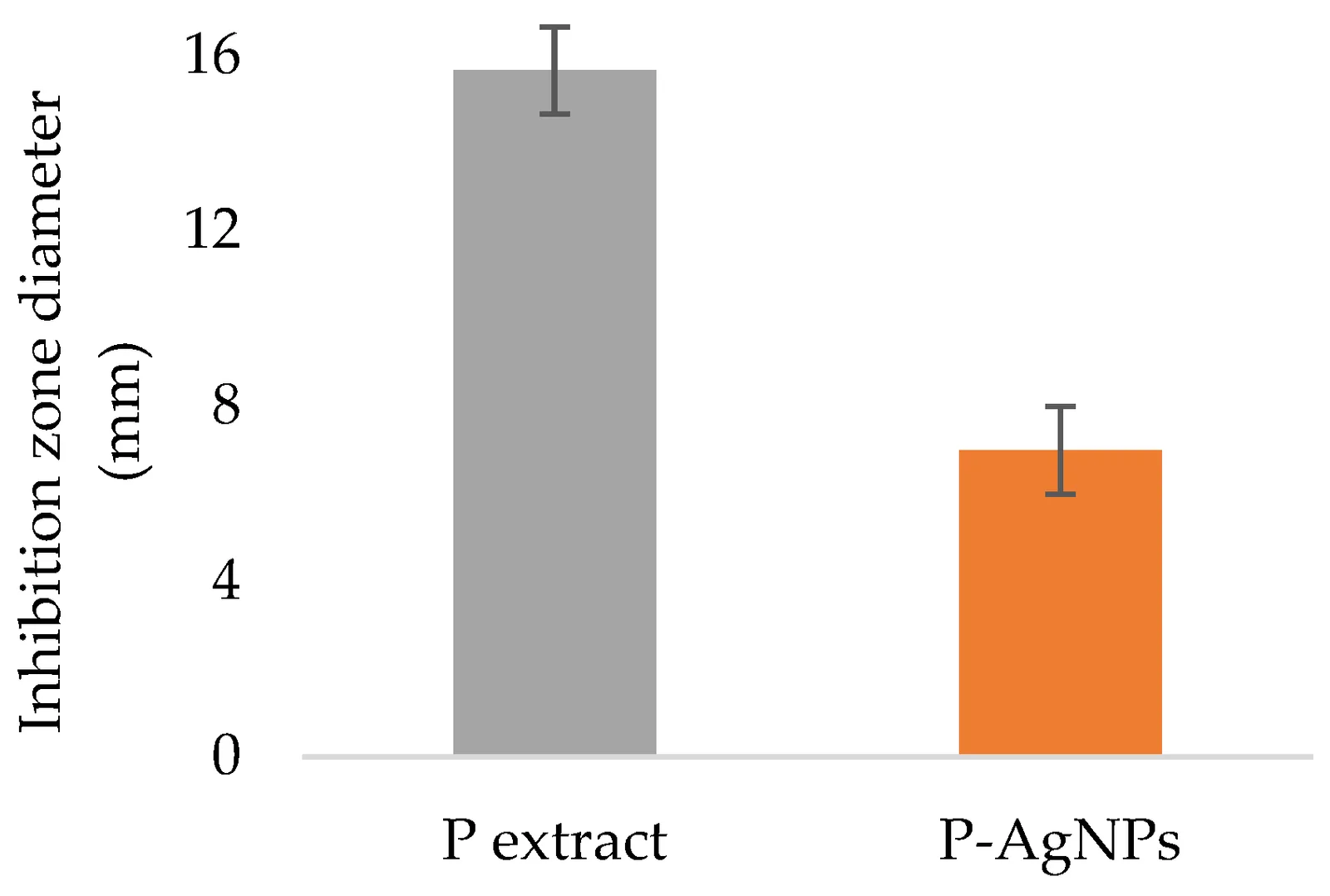
Average diameter of inhibition zones produced by plant extracts and the corresponding AgNP-containing phytosynthesis preparations against *Botrytis cinerea* ATCC 11542. Error bars represent standard deviation (*n* = 3).

Unlike the strains of *Penicillium hirsutum* and *Aspergillus niger*, *Botrytis cinerea* showed selective sensitivity to the investigated treatments. Of all the extracts and AgNP-containing phytosynthesis preparations tested, only the products obtained from orange peels generated measurable zones of inhibition. The average diameter of the zone of inhibition was 15.67 mm for the ethanolic extract of orange peels and 10.50 mm for the corresponding AgNP-containing phytosynthesis preparation (Figure 30). In the case of the other samples, no clear zones of inhibition were observed, suggesting a reduced or absent efficiency against this fungal species. The results indicate that the antifungal activity against *Botrytis cinerea* is strongly dependent on the nature of the plant extract used. Compared with the corresponding AgNP-containing phytosynthesis preparation, the ethanolic extract of orange peels showed a more pronounced inhibitory effect. However, given the small number of treatments that produced measurable inhibition zones, statistical interpretation should be performed with caution. These observations may be related to compositional changes occurring during the phytosynthesis process; however, the present experimental design does not allow the respective contributions of extract-derived compounds, residual Ag^+^, and nanoparticulate silver to be distinguished.

### 3.12. Activity Against Fusarium oxysporum ATCC 48112

**Figure 31:**
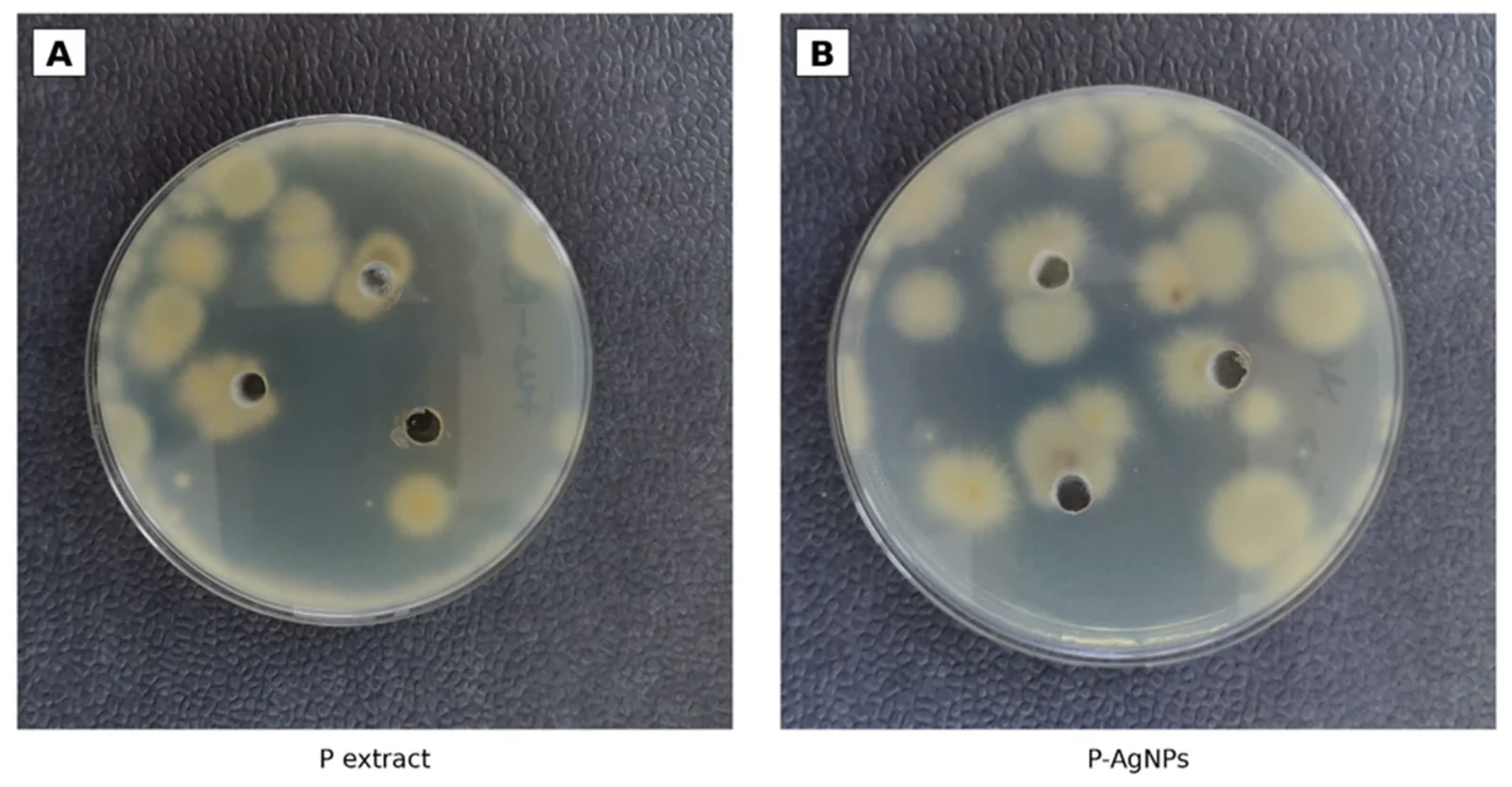
Representative images of the growth of *Fusarium oxysporum* ATCC 48112 strain on PDA medium after treatment with P extract (**A**) and P-AgNPs (**B**).

In the case of *Fusarium oxysporum*, the antifungal activity could not be quantitatively evaluated using the agar well diffusion method. The rapid radial growth and extensive mycelial development observed on PDA plates prevented the clear delimitation of reproducible inhibition zones around the wells. Consequently, inhibition diameters were not determined, and the results were recorded as ND (Not Determined). Given the rapid and invasive radial growth of *F. oxysporum*, which prevented reliable quantification using the agar well-diffusion assay, future studies should employ complementary quantitative approaches, such as broth microdilution or dry mycelial biomass inhibition assays, to more accurately assess the antifungal response of this strain. Representative images of the culture plates revealed that the fungal mycelium rapidly colonized the available surface, masking potential local effects of the tested extracts and phytosynthesized silver nanoparticles (Figure 31). Therefore, no quantitative conclusion regarding the susceptibility of *F. oxysporum* to the investigated treatments could be drawn under the experimental conditions employed.

The results obtained revealed that the antifungal efficiency of plant extracts and silver nanoparticles obtained by phytosynthesis was strongly dependent on the fungal species investigated. The highest sensitivity was observed in the case of the *Penicillium hirsutum* strain, for which the largest diameters of the inhibition zones were recorded, especially for ethanolic extracts from orange and pomegranate peels. In the case of the *Aspergillus niger* strain, although numerical differences between treatments were observed, these were not statistically confirmed. *Botrytis cinerea* presented a selective response, being sensitive only to treatments obtained from orange peels, while the rapid and extensive development of the *Fusarium oxysporum* mycelium prevented the quantitative evaluation of the inhibition zones by the method used.

Overall, the ethanolic extracts exhibited more pronounced antifungal activity than the corresponding AgNP-containing phytosynthesis preparations, indicating that conversion of the extracts into AgNP-containing preparations did not enhance antifungal activity under the conditions investigated.

These results confirm the potential of exploiting fruit peels as a source of compounds with antifungal activity and highlight the importance of selecting the treatment according to the target microorganism.

## 4. Discussion

### 4.1. Obtaining Extracts and AgNP Phytosynthesis

The selected plant materials obtained from the epicarp of *Punica granatum* L. (pomegranate), *Diospyros kaki* L. (persimmon), and *Citrus sinensis* Osbeck (orange), considered in some cases waste, have proven to be valuable biological resources for obtaining extracts rich in antioxidant compounds and with biological properties useful in plant disease management. The extracts and AgNP mixtures have the potential to stimulate physiological and biochemical parameters of *Triticum aestivum* L., *Zea mays* L., and *Cucumis sativus* L. test plants or inhibit the growth of weeds in various crops or landscaped spaces.

Our results confirm the high content of bioactive compounds, at least for some of the samples tested. The high content of phenolic compounds in pomegranate peel extract is correlated with a high antioxidant capacity [26]. Mostafa et al. (2021) [10] also observed a higher phenolic content in pomegranate peel extract (“an exorbitant amount of quercitrin, 23.62 mg g^−1^ d.w.”) than in orange (“a high amount of chlorogenic acid, 5.92 mg g^−1^ d.w.”).

The presence of phenolic compounds and other biomolecules, also confirmed in the FTIR spectrum, had an important role in the phytosynthesis of AgNPs as reducing and capping agents during green synthesis [27]. Collectively, the comparable FTIR spectral behavior observed for all three plant extracts supports a common green synthesis mechanism mediated by polyphenolic biomolecules [28,29]. The phytosynthesis process of silver nanoparticles was accompanied by visible changes in the color of the extracts and changes in the UV-Vis spectra, confirming the involvement of bioactive compounds in the reduction of Ag^+^ ions and the formation of nanoparticles. In the case of the orange extract, the color intensification to dark brown during incubation, as well as the appearance and increase in the intensity of plasmonic benzenes in the UV-Vis spectra, indicate the progressive formation of silver nanoparticles. These observations are consistent with the role of phenolic compounds and other plant metabolites as reducing agents and stabilizers in the green synthesis of nanoparticles.

Dimensions and UV-Vis spectra close to those obtained in the present research and a slight spectral shift in the obtained data after 3 days confirm the formation of AgNPs in the investigated extracts. For AgNPs with sizes from 8 to 14 nm obtained from pomegranate and orange peel extracts, average wavelengths of 437 nm and 450 nm were recorded [10]. AgNP synthesis was also confirmed by Bhat et al. (2024) [30] through UV-Vis spectrum, with peaks recorded at 400 nm for AgNPs from the extract of the epicarp of *C. sinensis* and at 430 nm for *C. limon*; Niluxsshun et al. (2021) [31] reported a peak at approximately 440 nm. AgNPs with dimensions of 20.7 ± 9.1 nm and 27.12 nm obtained from kaki extracts showed peaks at 411 nm [32] and 453 nm [9], and the spherical ones with sizes between 5 and 50 nm obtained from pomegranate peel showed a maximum absorption at 437 nm. 

The decrease in antioxidant capacity in extracts in which AgNPs were formed confirms the role of biologically active compounds in the phytosynthesis of nanoparticles. Mickky et al. (2024) [33] observed that the antioxidant capacity (free radical-scavenging ability, reducing power, and antioxidant activity) of the synthesized AgNPs was lower than that of the orange peel extract. In the literature, many opinions exist regarding the comparison between the antioxidant capacity of an extract with and without AgNPs. In general, the extract without AgNPs has a higher antioxidant capacity than those with NPs because the phytochemicals are often used for the reduction of Ag^+^ ions [34]. Abdel-Aziz et al. (2014) [35] consider that AgNPs do not contribute much to the antioxidant activity, the main role being held by the plant extract itself and more specifically by phenolic compounds which have redox properties. Rani et al. (2024) [36] reported that the DPPH radical scavenging activity of the pomegranate peel extract without and with AgNPs was dose-dependent, reaching maximal levels of 61.42% and 82.24%, respectively. The increased scavenging activity of AgNPs obtained from pomegranate peel (84.85–89.20%) [36] may be due to the presence of bioactive molecules on the AgNP surface [37].

For a synthetic comparison of the main phytochemical and antioxidant parameters of the plant extracts, the results were also represented in the form of a heatmap normalized by Z-score transformation (Table 5). The independent normalization of each parameter allowed the comparison of the relative profiles of the three extracts, eliminating the influence of differences between the units of measurement and the ranges of variation of the data. The heatmap highlights the fact that the pomegranate peel extract presents the highest content of phenolic compounds and the best preservation of antioxidant activity after phytosynthesis, while the orange peel extract is characterized by the most pronounced decrease in DPPH activity after obtaining silver nanoparticles. The persimmon peel extract presents an intermediate profile for most of the analyzed parameters.

### 4.2. Influence of Extracts and AgNP-Containing Phytosynthesis Preparations on Growth, Biomass, Pigment and Polyphenol Content in Triticum aestivum L., Zea mays L. and Cucumis sativus L.

The extraction of bioactive compounds from the peels of fruits had a positive effect on the growth of the three plant species of seeds compared with the variants with ethanol, in which no germination was recorded. Generally, the degree of inhibition/stimulation is more pronounced at the radicle level because roots are the first vegetative organs to develop, so their contact time with the substances from the environment is greater than that of shoots [38].

A summary of the significant changes observed following treatment with the crude extracts and the corresponding AgNP-containing phytosynthesis preparations in the three tested plant species is presented in Table 6. The most positive effects were induced in maize, *Zea mays* L., where no significant inhibitory effect was recorded. On the other hand, the most inhibitory effects (15) were obtained in cucumber, *Cucumis sativus* L., indicating the differential response of dicots (*Cucumis*) compared to monocots (*Zea* and *Triticum*).

Citrus peel, often considered waste, contains compounds (terpenoids, alkaloids, and phenols) with antioxidant and antimicrobial potential, thus supporting their valorization in the pharmaceutical and food industries, in accordance with the principles of sustainable use of natural resources [39]. The application of orange peel extract (600 mg L^−1^) significantly stimulated the growth, photosynthetic pigment content, and enzymatic activity of quinoa, highlighting its potential as a natural biostimulant in sustainable agriculture [40]. AgNPs phytobiosynthesized from orange peel extract improve faba bean plant growth under greenhouse conditions [41]. Athanasiadis et al. (2023) [6] confirmed that extracts obtained from persimmon peels using ecological extraction methods are effective biostimulants that can improve plant growth and resistance to stress factors. This approach promotes the sustainable recovery of agro-industrial waste, contributing to sustainable agriculture and environmental protection.

Research regarding the effect of AgNPs on plants remains highly inconsistent, with results varying drastically from severe inhibition to marked biostimulation [42]. AgNPs can inhibit seedling growth in different crops [43] by blocking metabolic reactions [44] or by inducing oxidative stress [45]. The comparisons between different species require a standardized protocol for AgNP exposure [44] because the effect of the treatment with AgNPs depends on the type of application and the properties of the nanoparticles used (size, concentration, synthesis method, coating), etc. [45].

Green AgNPs can enhance root and shoot development, fresh weight [46], germination, and crop yield without modifying the plant’s natural attributes [47]. AgNPs modulate plant development by directly interacting with cellular proteins, enzymes, and carbohydrates, which in turn alter plant growth regulator biosynthesis. These hormone systems regulate cell division and elongation [48].

AgNPs enhance chlorophyll concentrations by promoting water retention [49]. Furthermore, they stimulate biomass accumulation by driving nutrient uptake [50], which is facilitated by xylem opening, a mechanism that directly accelerates nutrient translocation throughout the plant [51]. They can also improve plant responses to biotic and abiotic stress and even increase the production of bioactive compounds and antioxidants.

Low concentrations of AgNPs elicited modest increases in the stem length and total fresh seedling mass of wheat seedlings [42], improved plant growth, PSII efficiency [52], and reduced leaf aging. AgNPs are important for minimizing abiotic and biotic stress in wheat by improving drought [53] and low-temperature tolerance, reducing ozone-induced damage [54], improving the response to salt stress, and increasing resistance to *Bipolaris sorokiniana* [55] without adversely affecting plant growth.

Hussain et al. (2024) [56] determined the highest germination index, root/shoot growth, and fresh/dry weights for *Zea mays* in AgNP-containing variants. The content of chlorophyll, carotenoids, and total soluble sugars was also enhanced by the application of nanoparticles. *Zea mays* seedling lengths and weights were stimulated by 10 ppm AgNPs even in hydric stress conditions (under 30% drought level). AgNPs improve maize germination during drought by triggering pre-germination metabolic activities [57]. Maize plant growth attributes were significantly enhanced by seed priming and foliar application of AgNPs, offering a sustainable strategy to mitigate chromium-induced toxicity [58]. AgNPs counteract seed aging and enhance vigor in sensitive maize lines by regulating stress response, metabolic repair, and membrane stability-related genes [59].

AgNPs had a positive impact on root and shoot length in rice [60], seed germination and seedling development in cucumber [48], and phytochemical pigment and phenol concentrations in tomato [61]. AgNPs are powerful elicitors that modulate plant metabolism [62] and activate antioxidant defenses by upregulating phenolic compound content [63].

### 4.3. Antifungal Activity of Extracts and AgNP-Containing Phytosynthesis Preparations

The results obtained suggest the existence of a relationship between the phytochemical composition of the extracts and their antifungal activity.

The ethanolic extract of pomegranate peels presented the highest total content of phenolic compounds (147.52 mg g^−1^ f.w.), followed by the orange peel extract (64.18 mg g^−1^ f.w.) and the persimmon peel extract (37.31 mg g^−1^ f.w.). The same trend was observed in the case of antifungal activity against *Penicillium hirsutum*, where the pomegranate extract generated the largest zone of inhibition (30 mm), followed by the orange extract (27 mm) and the persimmon extract (17.33 mm). The agreement between the two series of results suggests that phenolic metabolites contribute significantly to the antifungal effect of the investigated extracts.

Research in the field indicates the bioactive and antimicrobial potential of phytosynthesized AgNPs. Low concentrations of AgNPs stimulate cucumber root growth [14] and serve as an efficient biocontrol agent against powdery mildew in cucumber [64]. Orange peel-derived AgNPs exhibited remarkable inhibitory effects against chloramphenicol-resistant bacteria *Alcaligenes faecalis* and *Morganella morganii*, as well as against ketoconazole-resistant *Penicillium digitatum* and *Fusarium oxysporum* fungi [65].

A descriptive comparison between total phenolic content and antifungal activity revealed a similar trend for *P. hirsutum*. The pomegranate peel extract, which exhibited the highest total phenolic content (147.52 mg g^−1^ f.w.), produced the largest inhibition zone (30 mm), followed by the orange extract (64.18 mg g^−1^ f.w.; 27 mm) and the persimmon extract (37.31 mg g^−1^ f.w.; 17.33 mm). Thus, for *P. hirsutum*, the ranking of antifungal activity paralleled that of total phenolic content, suggesting a possible contribution of phenolic compounds to the observed inhibitory effect. However, this relationship was not consistently observed across the other tested fungal species. Therefore, total phenolic content alone cannot explain the antifungal activity of the investigated extracts, which may also depend on their specific phytochemical composition and on the susceptibility of the target fungal species. Given the limited number of extracts investigated (*n* = 3), a formal statistical correlation analysis was not considered sufficiently robust. Comparison of the activity of the crude extracts with that of the corresponding AgNP-containing phytosynthesis preparations revealed that the crude extracts generally produced larger inhibition zones. For example, the diameter of the inhibition zone against *Penicillium hirsutum* decreased from 30 mm to 18.33 mm for pomegranate, from 27 mm to 15.67 mm for orange, and from 17.33 mm to 13.67 mm for persimmon. Similarly, the antioxidant activity determined by the DPPH assay decreased after the phytosynthesis process, with the largest decrease observed for the orange extract (90.72% → 35.17%). These changes may be associated with the involvement of antioxidants and reducing compounds during the phytosynthesis process. However, because the individual contributions of extract-derived compounds, residual Ag^+^, and nanoparticulate silver were not separately determined, the mechanisms underlying the observed changes cannot be established from the present data.

The differences in sensitivity observed between fungal species indicate that the biological response is influenced both by the characteristics of the extracts and by the particularities of each fungal species. *Penicillium hirsutum* was the most sensitive species, while *Aspergillus niger* showed a lower sensitivity, and *Botrytis cinerea* responded selectively only to orange-based treatments. These results suggest that antifungal efficacy is determined by both the number of bioactive compounds and their specific composition.

The results demonstrate that extracts with a higher content of phenolic compounds and high antioxidant activity also showed superior antifungal activity, while the phytosynthesis process partially modified these properties by involving plant metabolites in the reduction and stabilization of silver nanoparticles. These observations highlight the potential dual role of phytochemicals in both the green synthesis process and the biological activity of the resulting preparations.

The agar well diffusion assay used in the present study provides a comparative assessment of fungal growth inhibition under the tested conditions but does not allow determination of MIC or MFC values or a complete concentration-dependent characterization of antifungal potency. Therefore, the present results should be interpreted as comparative screening data. Further studies employing broth-based MIC/MFC determinations, mycelial growth inhibition assays, or growth-kinetic approaches would be required to quantitatively characterize the antifungal potency of the investigated systems.

An important limitation of the present study is that the biological assays were performed using the complete phytosynthesis preparations. Therefore, the individual contributions of nanoparticulate silver, residual Ag^+^, and extract-derived bioactive compounds to the observed effects cannot be distinguished. Further studies involving purified and redispersed AgNPs, reaction supernatants, appropriate AgNO_3_ controls, and quantitative determination of ionic and nanoparticulate silver fractions are required to elucidate their respective contributions to the biological activity.

Future compositional studies using GC-MS and/or complementary chromatographic techniques could enable the identification and quantification of individual bioactive compounds and provide further insight into their contribution to the observed biological activity.

## 5. Conclusions

This study provided a comparative evaluation of pomegranate, orange, and persimmon peel extracts and their corresponding AgNP-containing phytosynthesis preparations. The three fruit peel extracts contained bioactive compounds capable of participating in AgNP phytosynthesis, as supported by UV-Vis, FTIR, BF-STEM, and EDS analyses. The resulting nanoparticles differed in their morphological and dimensional characteristics depending on the plant source.

The biological responses varied according to the plant species and treatment tested. Under the experimental conditions employed, maize showed lower overall sensitivity to the investigated treatments, whereas wheat and cucumber exhibited more pronounced treatment-dependent changes in growth and biochemical parameters.

The antifungal screening showed a fungal-species-dependent response. *Penicillium hirsutum* exhibited the greatest sensitivity among the tested strains, particularly to pomegranate and orange peel extracts, whereas *Aspergillus niger* showed a more variable response and *Botrytis cinerea* responded only to the orange-derived treatments. Quantitative inhibition could not be established for *Fusarium oxysporum* using the agar diffusion assay employed. Importantly, the AgNP-containing phytosynthesis preparations did not consistently produce greater antifungal effects than the corresponding crude extracts.

The results support the value of fruit peels as sources of bioactive compounds and as reducing and stabilizing matrices for AgNP phytosynthesis. However, the present findings should be interpreted within the limitations of the experimental design. Further studies using purified nanoparticle fractions, silver-speciation analysis, and concentration-dependent antifungal assays are required before the specific contribution of nanoparticulate silver and the potential applicability of these systems in plant protection can be established.

## Figures and Tables

**Table 1:** Experimental variants for phytotoxicity tests.

Variants	Contents	Dilution
Control	distilled water	-
Ethanol	ethanol 96%	-
K10	ethanolic extract of persimmon (*Diospyros kaki* L.) peel	10
K100		100
KNP10	ethanolic extract of persimmon peel with AgNPs	10
KNP100		100
P10	ethanolic extract of orange (*Citrus sinensis* Osbeck) peel	10
P100		100
PNP10	ethanolic extract of orange peel with AgNPs	10
PNP100		100
R10	ethanolic extract of pomegranate (*Punica granatum* L.) peel	10
R100		100
RNP10	ethanolic extract of pomegranate peel with AgNPs	10
RNP100		100

**Table 2:** Positions of FTIR absorption band maxima for fruit extracts functionalized with silver nanoparticles.

Samples (cm^−1^)						Attribution
K	KPN	P	PNP	R	RNP	
3264	3240	3326	3250	3313	3257	O–H stretching (phenols)
2977 2932		2972 2927 2885		2973 2929 2885		C-H stretching (asym CH_3_ and CH_2_) C-H stretching (asym CH_3_)
1645	1636	1647	1636	1645	1635	C=O stretching
	1558–1507		1540–1507		1540–1508	amide II
1383		1379 1326	1311	1380 1330	1342	C–N and COO^−^ vibrations
1082 1042	1077 1044	1086 1044	1085 1045	1085 1043	1042	C–O stretching
876		879		878		Aromatic C–H

**Table 3:** Antioxidant activity of extracts and AgNP mixtures.

Variants	Inhibition Percentage (%)
R	86.23
RNP	83.18
P	90.72
PNP	35.17
K	91.20
KNP	55.42

**Table 4:** Heatmap of the antifungal effect of plant extracts and AgNP mixtures against the tested fungal strains.

Fungal Strain	Experimental Variants					
	P	R	K	PNP	RNP	KNP
*Penicillium hirsutum* ATCC 52323						
*Aspergillus niger* ATCC 15475						
*Botrytis cinerea* ATCC 11542						
*Fusarium oxysporum* ATCC 48112	ND	ND	ND	ND	ND	ND

ND = not determined. Background colors represent the magnitude of the antifungal effect, with increasing color intensity from light yellow to red corresponding to increasing inhibition zone diameters.

**Table 5:** Normalized heatmap (Z-score) of total phenolic content and antioxidant activity of ethanolic fruit peel extracts.

Parameter	K	P	R
Total phenolic content			
DPPH (extract)			
DPPH (AgNPs)			
DPPH decrease after AgNP phytosynthesis			

For each parameter, the values were independently normalized by Z-score transformation. The background colors represent the normalized values: shades of red indicate values above the mean of the analyzed parameter, while shades of blue indicate values below the mean.

**Table 6:** Significant changes observed following treatment with extracts and AgNP-containing phytosynthesis preparations in the tested plants.

Biological Parameter	Experimental Variants																									
	C		K10		K100		KNP10		KNP100		P10		P100		PNP10		PNP100		R10		R100		RNP10		RNP100	
	*Triticum aestivum* L. Significant Changes																									
	+	−	+	−	+	−	+	−	+	−	+	−	+	−	+	−	+	−	+	−	+	−	+	−	+	−
Root length					+				+				+		+		+			−	+				+	
Stem length					+															−					+	
Fresh weight													+				+									
Polyphenols			+					−					+													
Pigments																	+									
	*Zea mays* L. significant changes																									
Root length			+		+		+		+		+		+		+		+		+		+		+		+	
Stem length			+		+		+		+		+		+		+		+		+		+		+		+	
Fresh weight			+		+				+		+		+		+		+		+				+			
Polyphenols			+																							
Pigments																	+		+							
	*Cucumis sativus* L. significant changes																									
Root length				−				−				−				−				−				−		−
Stem length																	+								+	
Fresh weight				−		−						−				−								−		−
Polyphenols																										
Pigments				−														−								
TOTAL	+	−	+	−	+	−	+	−	+	−	+	−	+	−	+	−	+	−	+	−	+	−	+	−	+	−
			5	3	5	1	2	2	4	0	3	2	6	0	4	2	8	1	4	3	3	0	3	2	5	2

“+” indicates a positive effect, while “−” indicates a negative effect compared with the control.

## Data Availability

The original contributions presented in this study are included in the article/Supplementary Material. Further inquiries can be directed to the corresponding author.

## Supplementary Materials

The following supporting information can be downloaded at: https://www.mdpi.com/article/10.3390/ma19163492/s1, Figure S1: ATR-FTIR of the ethanolic extract of *Citrus sinensis* Osbeck (A) and its AgNPs (B); Figure S2: ATR-FTIR of the ethanolic extract of *Punica granatum* L. (A) and its AgNPs (B); Figure S3: *Triticum aestivum* L. seedlings, 7 days after seed immersion; Figure S4: *Zea mays* L. seedlings, 7 days after seed immersion; Figure S5: *Cucumis sativus* L. seedlings, 7 days after seed immersion.

## Abbreviations

The following abbreviations are used in this manuscript:

AgNPs	Silver nanoparticles
ATR-FTIR	Attenuated total reflectance Fourier transform infrared spectroscopy
BF-STEM	Bright-field scanning transmission electron microscopy
DPPH	2,2-diphenyl-1-picrylhydrazyl
EDS	Energy-dispersive X-ray spectroscopy
fw	Fresh weight
GAE	Gallic acid equivalent
min	Minute(s)
PDA	Potato dextrose agar
TPC	Total phenol content
UV-Vis	Ultraviolet-visible spectroscopy
