# Supplementary material for: Exotic Fruit Peel Extracts as Green Resources for Bioactive Silver-Based Materials with Plant Protection Potential
Source: Materials (Basel). 2026 Aug 18;19(16):3492. doi: 10.3390/ma19163492 (PMC13514621; doi:10.3390/ma19163492)
Supplement: Supplementary file 1 [file materials-19-03492-s001.zip › materials-4474940-supplementary.pdf]

Exotic Fruit Peel Extracts as Green Resources for Bioactive Silver-Based Materials with Plant Protection Potential

Oana-Alexandra Luțu [1], Bianca-Maria Neagoe [1], Camelia Ungureanu [2,*], Codruța Mihaela Dobrescu [1], Loredana Elena Vijan [1], Carmen Mihaela Topală [1], Georgiana Cîrstea [3], Aurelian Denis Negrea [3], Nicoleta Anca Șuțan [1], Alina Păunescu [1], Elena Simona Pisculungeanu [1], Constanța Bucăloiu [1] and Liliana Cristina Soare [1]

[1] Faculty of Sciences, Physical Education and Computer Science, Pitesti University Centre, National University of Science and Technology Politehnica Bucharest, 1st Targu din Vale Str., 110040 Pitesti, Romania; oana.draghiceanu@upb.ro (O.-A.L.); bianca.smarandoiu@stud.sefi.upb.ro (B.-M.N.); codruta.dobrescu@upb.ro (C.M.D.); loredana.vijan@upb.ro (L.E.V.); carmen.topala@upb.ro (C.M.T.); nicoleta_anca.sutan@upb.ro (N.A.Ș.); alina.paunescu@upb.ro (A.P.); pisculungeanue@gmail.com (E.S.P.); mirelabucaloiu3@gmail.com (C.B.); liliana.soare@upb.ro (L.C.S.)

[2] Faculty of Chemical Engineering and Biotechnology, National University of Science and Technology Politehnica Bucharest, 313 Splaiul Independentei, Sector 6, 060042 Bucharest, Romania

[3] Regional Research and Development Center for Innovative Materials, Processes and Products for the Automotive Industry (CRC&D-Auto), Pitesti University Centre, National University of Science and Technology Politehnica Bucharest, 1st Targu din Vale Str., 110040 Pitesti, Romania; georgiana.cirstea93@upb.ro (G.C.); aurelian.negrea@upb.ro (A.D.N.)

* Correspondence: camelia.ungureanu@upb.ro

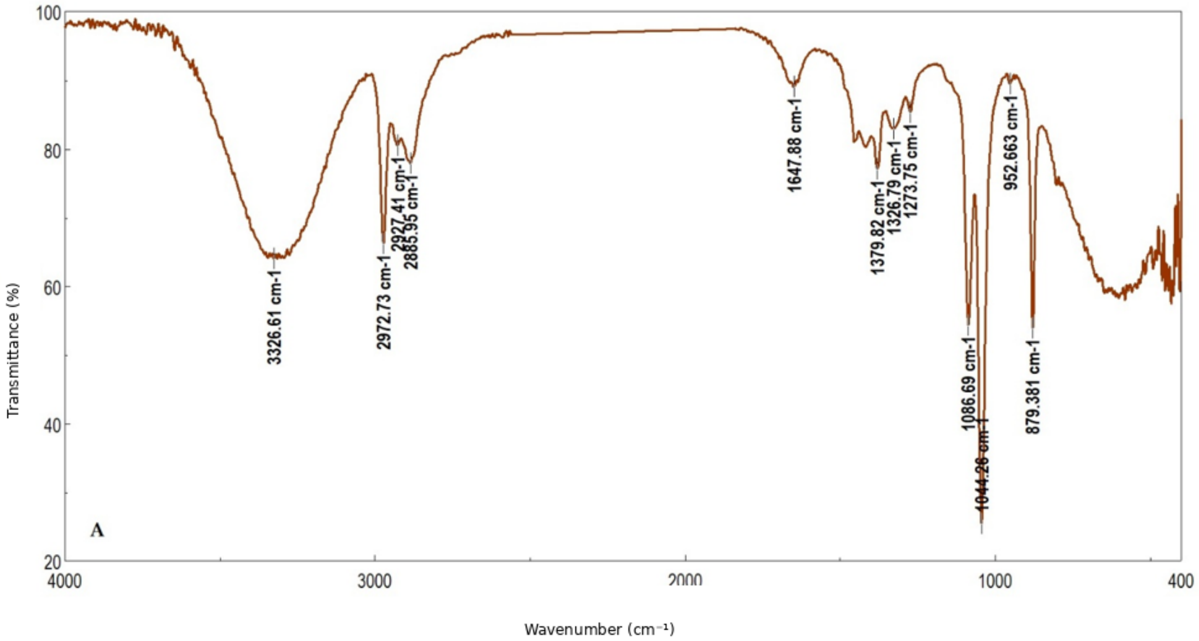

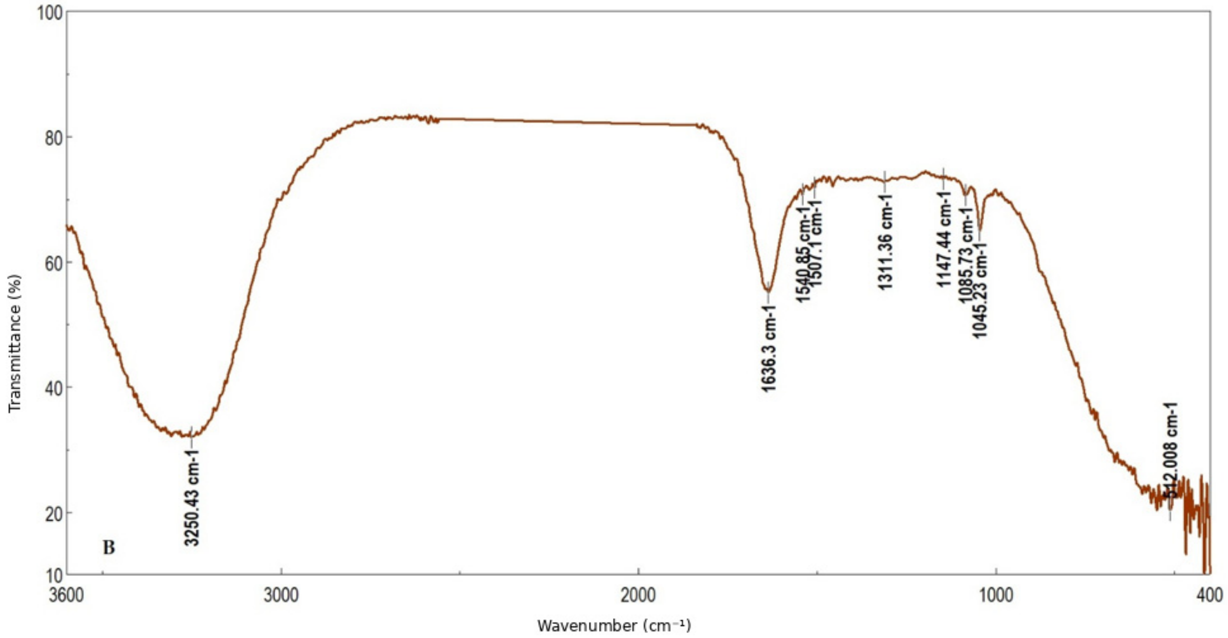

Figure S1. ATR-FTIR of the ethanolic extract of *Citrus sinensis* Osbeck (A) and its AgNPs (B)

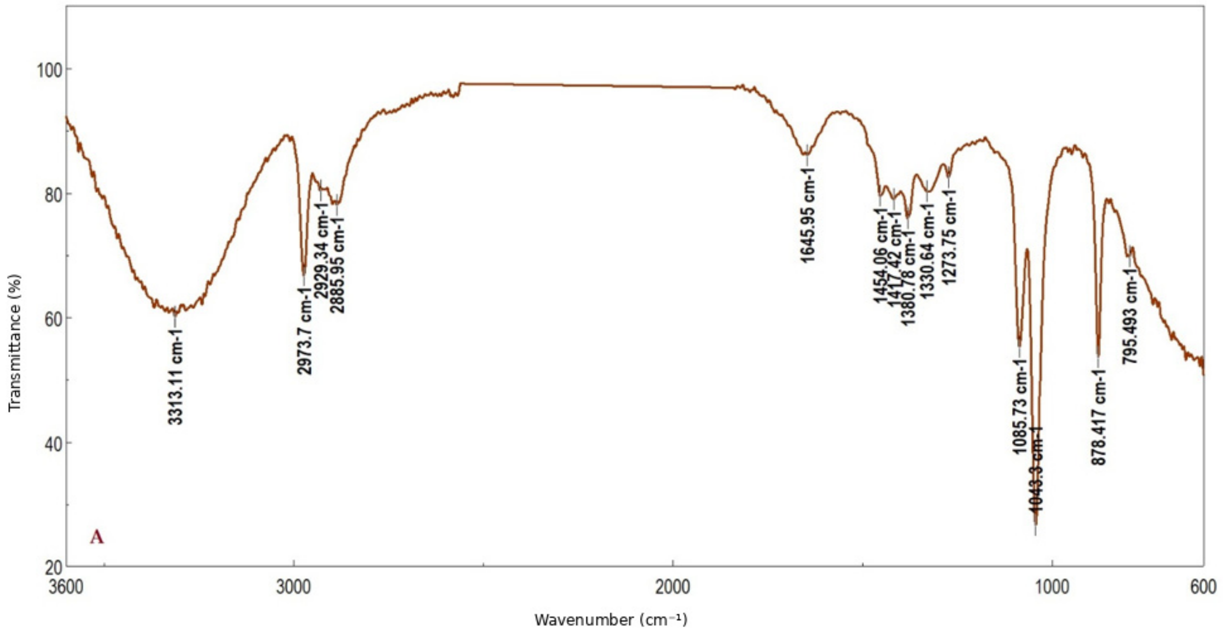

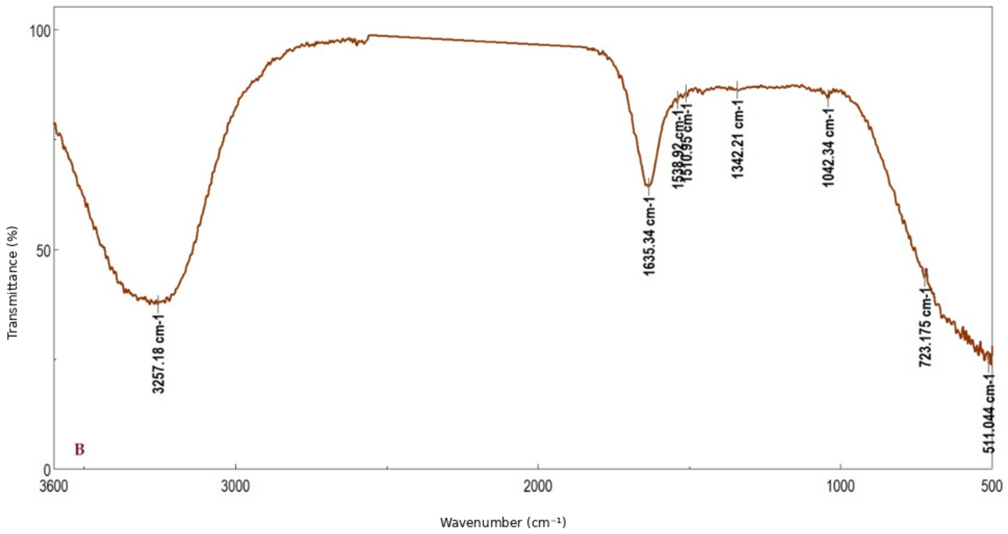

Figure S2. ATR-FTIR of the ethanolic extract of *Punica granatum* L. (A) and its AgNPs (B)

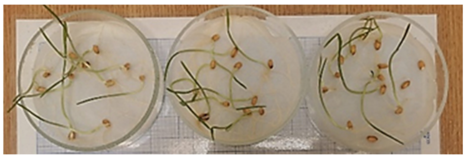

Control

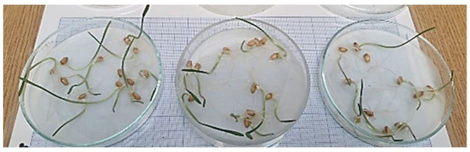

K10

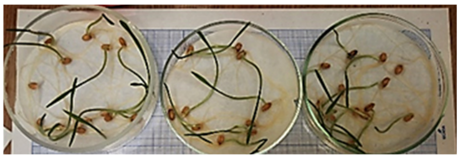

K100

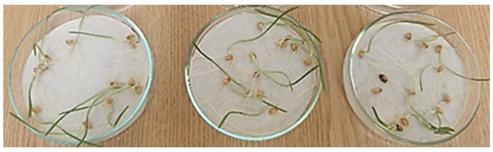

KNp10

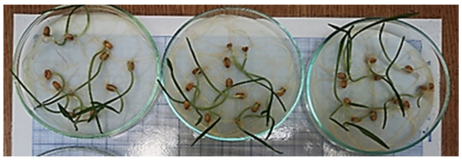

KNp100

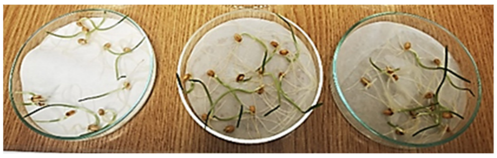

P10

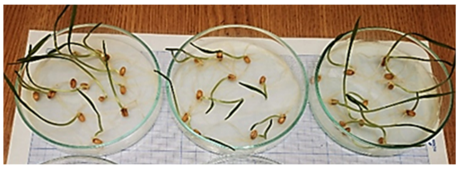

P100

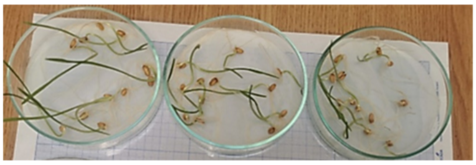

PNp10

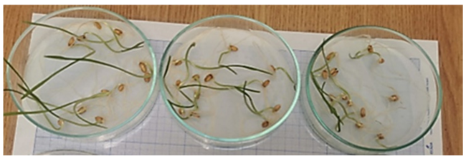

PNp100

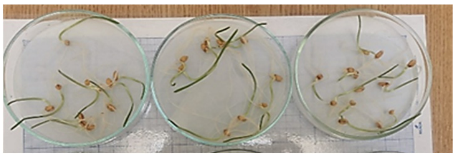

R10

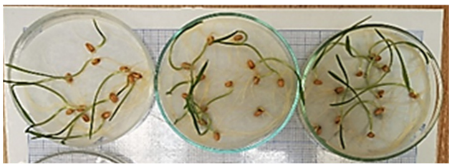

R100

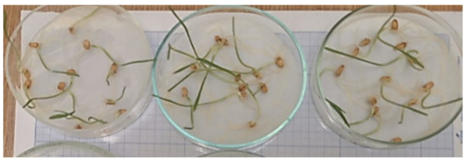

RNp10

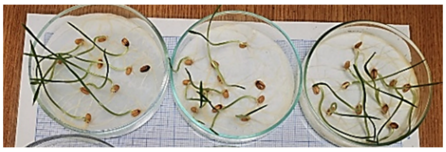

RNp100

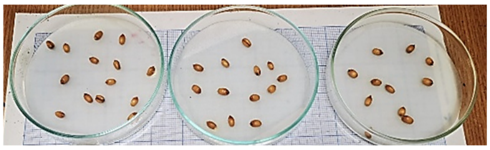

Ethanol

Figure S3. *Triticum aestivum* L. seedlings, 7 days after seed immersion

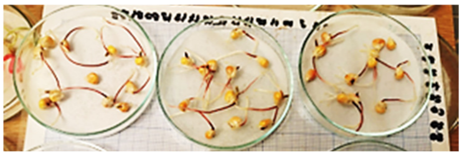

Control

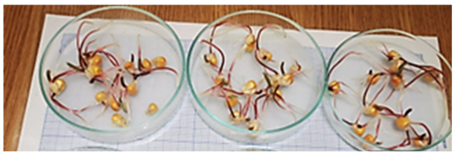

K10

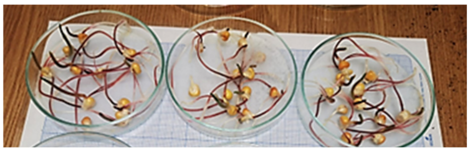

K100

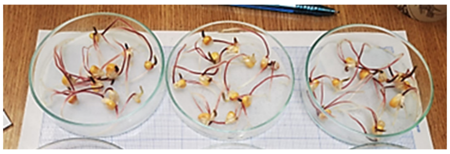

KNp10

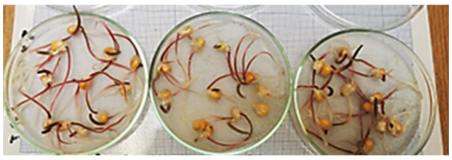

KNp100

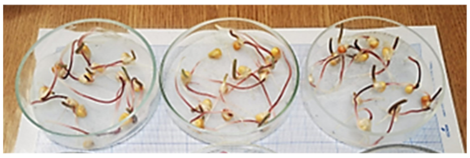

P10

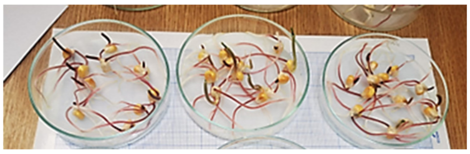

P100

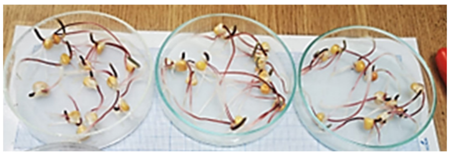

PNp10

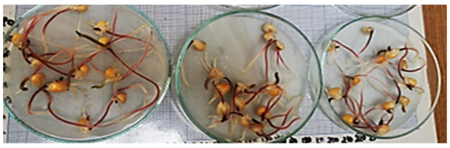

PNp100

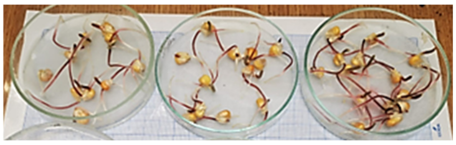

R10

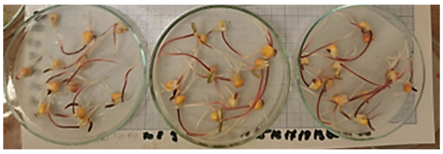

R100

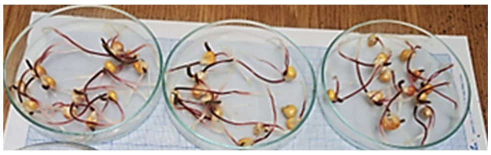

RNp10

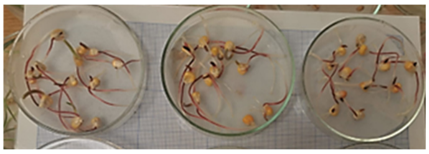

RNp100

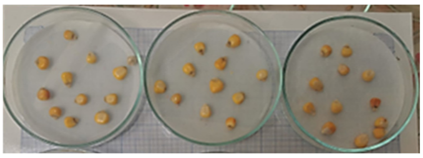

Ethanol

Figure S4. *Zea mays* L. seedlings, 7 days after seed immersion

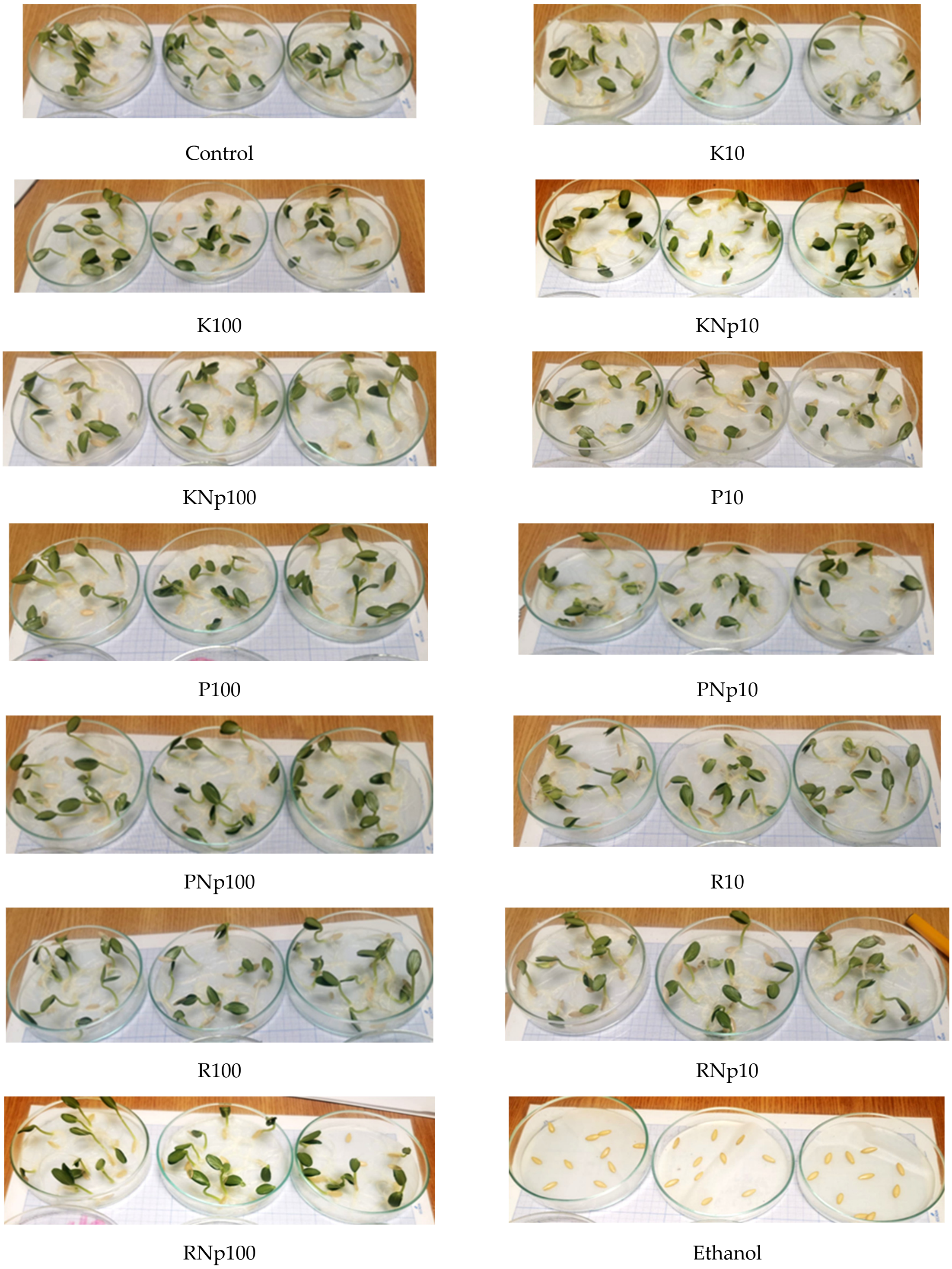

Figure S5. *Cucumis sativus* L. seedlings, 7 days after seed immersion
